# Improvement of the Proton Conduction of Copper(II)-Mesoxalate Metal–Organic Frameworks by Strategic Selection of the Counterions

**DOI:** 10.1021/acs.inorgchem.2c01241

**Published:** 2022-07-15

**Authors:** Beatriz Gil-Hernández, Simon Millan, Irina Gruber, Miguel Quirós, David Marrero-López, Christoph Janiak, Joaquín Sanchiz

**Affiliations:** † Departamento de Química, Facultad de Ciencias, Sección Química, Universidad de La Laguna, La Laguna 38206, Tenerife, Spain; ‡ Institute of Materials and Nanotechnology, Universidad de La Laguna, P.O. Box 456, La Laguna E-38200, Tenerife, Spain; § Institut für Anorganische Chemie und Strukturchemie, Heinrich-Heine Universität Düsseldorf, 40204 Düsseldorf, Germany; ∥ Departamento de Química Inorgánica, Facultad de Ciencias, Universidad de Granada, 18071 Granada, Spain; ⊥ Departamento de Física Aplicada I, Universidad de Málaga, Campus Teatinos s/n, 29071 Málaga, Spain

## Abstract

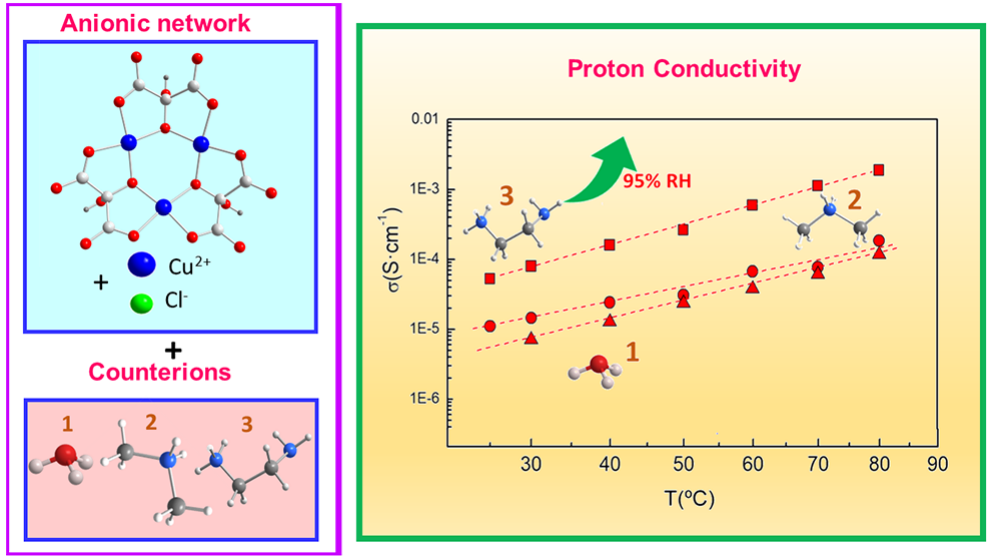

Three copper(II)/mesoxalate-based MOFs with formulas (H_3_O)[Cu_9_(Hmesox)_6_(H_2_O)_6_Cl]·8H_2_O (**1**), (NH_2_Me_2_)_0.4_(H_3_O)_0.6_[Cu_9_(Hmesox)_6_(H_2_O)_6_Cl]·8H_2_O (**2**), and (enH_2_)_0.25_(enH)_1.5_[Cu_6_(Hmesox)_3_(mesox)(H_2_O)_6_Cl_0.5_]Cl_0.5_·5.25H_2_O (**3**) were synthesized (H_4_mesox = mesoxalic acid = 2,2-dihydroxypropanedioic acid, en = ethylenediamine). Essentially, all of the compounds display the same anionic network with a different arrangement of the cations, which have a remarkable effect on the proton conduction of the materials, ranging from 1.16 × 10^–4^ S cm^–1^ for **1** to 1.87 × 10^–3^ S cm^–1^ for **3** (at 80 °C and 95% RH). These compounds also display antiferromagnetic coupling among the copper(II) ions through both the carboxylate and alkoxido bridges. The values of the principal magnetic coupling constants were calculated by density functional theory (DFT), leading to congruent values that confirm the predominant antiferromagnetic nature of the interactions.

## Abstract 2

Three copper(II)-mesoxalate metal−organic frameworks were synthesized in the presence of three different cations: hydronium, dimethylammonium, and ethylenediammonium, which neutralize the charge of the anionic networks. Besides the crystallographic characterization and the investigation of the magnetic properties, the compounds show varying proton conductivities depending on the included cations. The proton conductivity increases 1 order of magnitude in the case of compound **3** (1.87 × 10^−3^ S cm^−1^ at 80 °C and 95% RH), which contains ethylenediammonium cations.

## Introduction

Metal–organic frameworks (MOFs) are metal–ligand coordination networks that have gained much attention in the last years due to their porous properties and potential applications.^1−5^ The almost unlimited combinations of organic ligands and metal ions allow plenty of MOFs with physical and chemical properties such as magnetism, luminescence, electronic/ionic conduction, catalytic properties, and porosity with potential use in electronic or energy applications.^6−14^

In particular, MOFs as electrolytes in proton-exchange membrane fuel cells (PEMFCs)^14−16^ have recently attracted much interest, reaching ion conductivity values up to 10^–2^ S cm^–1^ under humid conditions^16−18^ comparable to the state-of-the-art Nafion electrolyte.^19,20^ MOFs have demonstrated a good performance in a broad temperature range, and their porous crystalline structure allows us to establish a mechanism for ionic conduction.

Moreover, the versatility of MOFs allows the combination of various physical/chemical properties in the same material, which is of great interest if interference between the different properties is achieved. In particular, the combination of conductive and magnetic properties has raised much interest due to their potential applications in electronics and spintronics.^21−30^

One strategic approach to achieve a metal–organic framework with magnetic and ionic conducting properties involves ensembling paramagnetic anionic networks with mobile cations in the pores. Evidently, the nature of the paramagnetic ions, the bridging ligands, and the mobility of the cations will be responsible for the magnetic and conducting properties of the material. In this sense, the combination of 3d paramagnetic ions, carboxylate bridging ligands, such as oxalate and derivatives, and protonated organic bases has been a very suitable approach.^22,23,25,28,29,31−43^

Our research group has previously reported that the mesoxalate ligand is a good candidate for obtaining functional materials.^44−54^ We have found that it can build one-dimensional (1D), two-dimensional (2D), and three-dimensional (3D) anionic paramagnetic networks, which can host water molecules and protonated organic bases in the pores, resulting in magnetic and proton-conducting materials.^14−23^

As an example of the success of this strategy, we have published the structure and properties of the compound {(H_3_O)[Cu_7_(Hmesox)_5_(H_2_O)_7_]·9H_2_O*_n_*, which exhibited long-range magnetic ordering with a *T*_C_ of 17 K and a conductivity of 6.5 × 10^–5^ S cm^–1^ at 23 °C and 100% of relative humidity (RH).^54^

As a continuation of that strategy, herein, we present three mesoxalate-copper(II) compounds that display conducting and magnetic properties, namely, (H_3_O)[Cu_9_(Hmesox)_6_(H_2_O)_6_Cl]·8H_2_O (**1**), (NH_2_Me_2_)_0.4_(H_3_O)_0.6_[Cu_9_(Hmesox)_6_(H_2_O)_6_Cl]·8H_2_O (**2**), and (enH_2_)_0.25_(enH)_1.5_[Cu_6_(Hmesox)_3_(mesox)(H_2_O)_6_Cl_0.5_]Cl_0.5_·5.25H_2_O (**3**).

We study the effect of the crystal lattice and its repercussion on the magnetic and conducting properties of the different cations (hydronium, dimethylammonium, and ethylenediammonium) hosted in the copper(II)/mesoxalate networks.

## Results and Discussion

### Synthesis and Characterization Studies

**Scheme 1:**
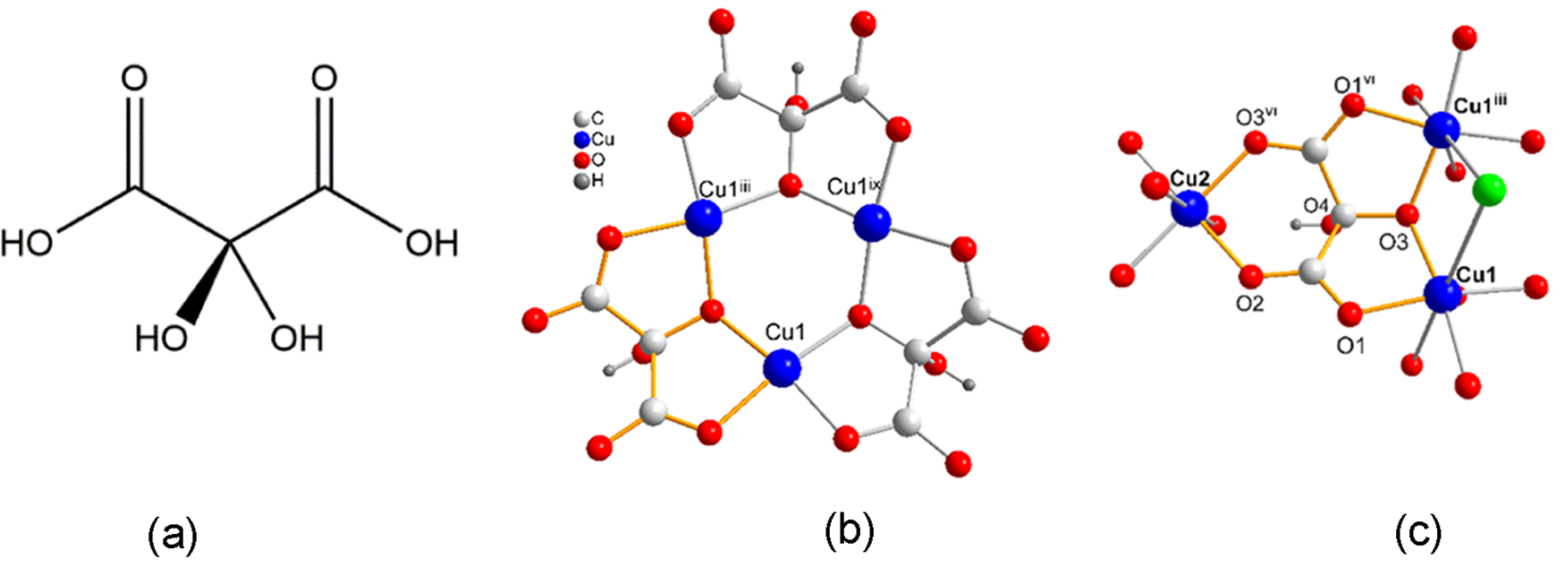
(a) Mesoxalic Acid, H_4_mesox. (b) Trinuclear Copper(II)-Mesoxalate Entity [Cu_3_(Hmesox)_3_]^3–^. (c) Coordination Mode μ_3_-Mesoxalate μ_3_-(κO:κO′,κO″:κO″,κO‴′:κO‴″) Symmetry codes: (iii) −*y*, *x* – *y*, *z*; (vi) −*y*, −*x*, *z*; (ix) −*x* + *y*, −*x*, *z*.

The reaction of basic copper(II) carbonate with an aqueous solution of mesoxalic acid (Scheme 1a) at 30 °C yields acidic solutions (pH ∼ 2.0) containing the [Cu_3_(Hmesox)_3_]^3–^ trinuclear species (Scheme 1b) in which the mesoxalic acid (H_4_mesox) has lost three of its four protons.^44−51^ These trinuclear copper(II) units further react with copper(II) and chloride ions (arising from copper(II) chloride tetrahydrate) to form anionic two-dimensional networks with general formula [Cu_9_(Hmesox)_6_Cl(H_2_O)_6_]*_n_*^*n–*^.

The additions of organic bases necessarily increase the pH to about 3 and provide the suitable cations to grow and balance the charge of the anionic network. For such a purpose, tetrabutylammonium hydroxide, dimethylamine, and ethylenediamine were added to obtain compounds **1**, **2**, and **3**, respectively. Hydronium and dimethylammonium cations were incorporated in (NH_2_Me_2_)_0.4_(H_3_O)_0.6_[Cu_9_(Hmesox)_6_(H_2_O)_6_Cl]·8H_2_O (**2**); however, in **1**, only H_3_O^+^ cations were incorporated instead of tetrabutylammonium, probably because of its volume, giving rise to (H_3_O)[Cu_9_(Hmesox)_6_(H_2_O)_6_Cl]·8H_2_O (**1**). Tetrabutylammonium cations were neither observed in the resolution of the structure nor the butyl group’s peaks found in the IR spectrum (Figure S1). Moreover, the elemental analysis did not fit with the occurrence of that cation. We postulate that the synthetic strategy of using the bulky tetrabutylammonium hydroxide is a successful method to obtain the reference material containing only hydronium counterions. This strategy was also used by our group to synthesize the coordination polymer {(H_3_O)[Cu_7_(Hmesox)_5_(H_2_O)_7_]·9H_2_O}*_n_*.^49^

The formula of compound **3** resulted in (enH_2_)_0.25_(enH)_1.5_[Cu_6_(Hmesox)_3_(mesox)(H_2_O)_6_Cl_0.5_]Cl_0.5_·5.25H_2_O, where enH^+^ and enH_2_^2+^counterions were incorporated. It has to be noted that ethylenediamine is mainly present as enH_2_^2+^ in the reaction medium (p*K*_a_1__ = 6.8 and p*K*_a_2__ = 9.9), but in the solid state, we have found that ethylenediamine is mainly monoprotonated, which satisfies the charge balance requirements of the anionic network and also agrees to the elemental analysis.

The purity of **1–3** was confirmed by positive matching of their experimental powder X-diffraction patterns with the simulated ones obtained from single-crystal diffraction data (Figure S3).

The thermogravimetric study shows mass loss percentages of 9.9, 8.8, and 7.3 wt % in the temperature range 20–150 °C for **1–3**, respectively, which correspond roughly to losses of 8, 8, and 5 crystallization water molecules (Figure S2). A progressive weight loss occurs above 150 °C, which corresponds to the loss of coordination water molecules together with the decomposition of the organic part of the material.

### Crystal Structures of **1–2**

**Figure 1:**
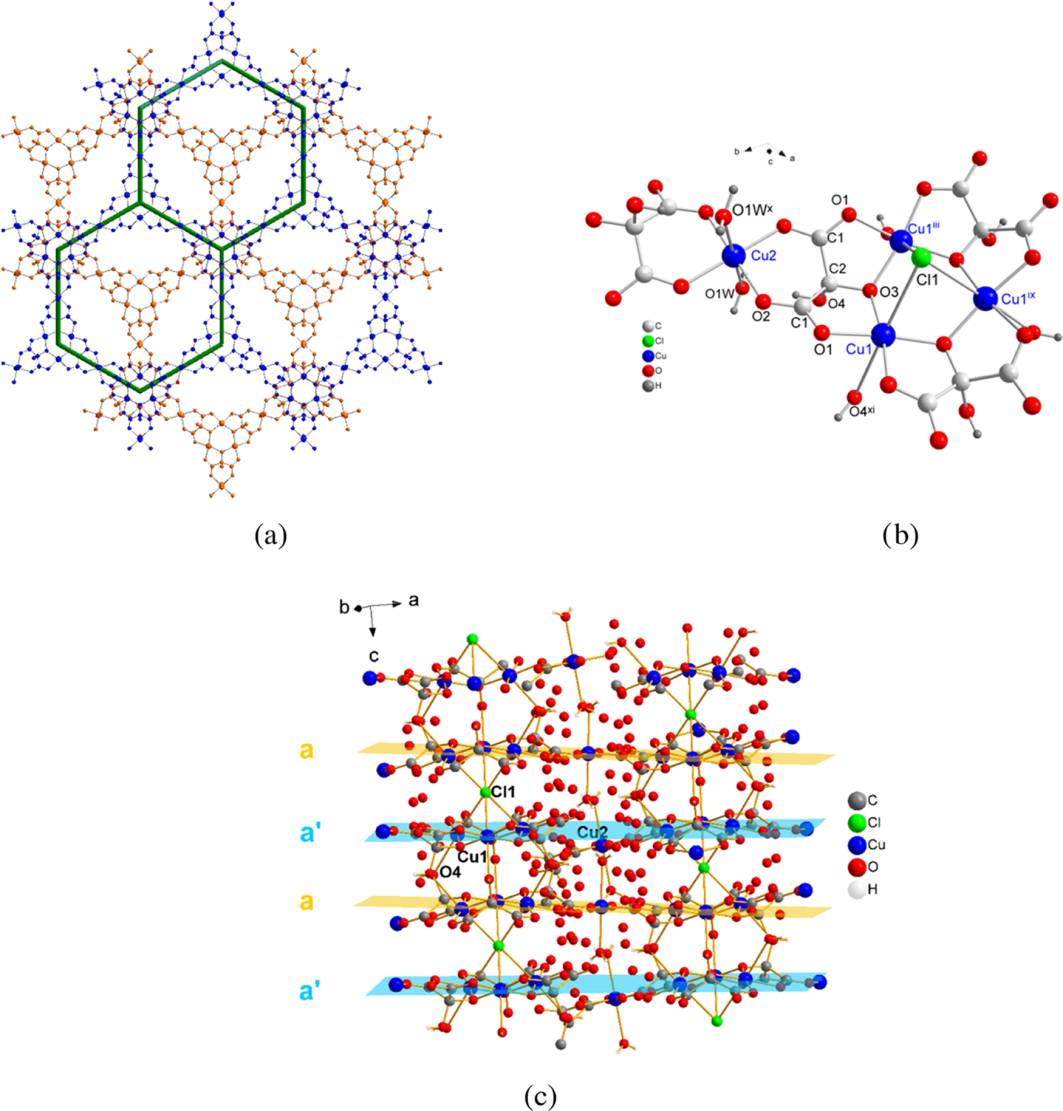
(a) View of the hexagonal anionic network for compound **1** along the *c* axis, showing in blue and in orange the layers a and a′ related by an inversion center. A hexagon connecting the Cu3 centroids of neighbor trinuclear copper(II) units is depicted showing the (6,3) topology of each single layer. (b) Coordination environments for Cu1 and Cu2 in **1**. (c) View of the stacking of a and a′ layers. Symmetry codes: (iii) −*y*, *x* – *y*, *z*; (ix) −*x* + *y*, −*x*, *z*; (x) *y*, *x*, −*z*; (xi) *x* – *y*, −*y*, −*z*.

Compounds **1** and **2** are isostructural (Figures S1 and S3) and crystallize in the hexagonal **R**3̅**m** space group (Figure 1 and Table S1). The structure builds up through stacking of two-dimensional hexagonal (6,3) anionic double layers A which stack in an AAAA manner to give a three-dimensional network with the cations (hydronium H_3_O^+^ for **1** and hydronium and dimethylammonium for **2**) and crystallization water molecules in the voids (Figure 1). These hexagonal layers connect to each other through large μ_6_-Cl bridges with a Cu1–Cl1 distance of 2.920 Å so that the structure is strictly three-dimensional, but for the seek of simplicity, we are considering it as essentially two-dimensional. A is a double layer that is composed of two single hexagonal layers, a and a′ related by an inversion center with (6,3) topology if we consider the Cu3 centroids as nodes (Figure 1a).

Both a and a′ single layers contain two kinds of crystallographic independent copper(II) ions, Cu1, Cu2, and one mesoxalate ligand (Figure 1).

Three symmetry-equivalent Cu1 atoms and three mesoxalate ligands are involved in copper(II)-mesoxalate trinuclear units with equilateral-triangular topology that work as three connectors (Scheme 1b and Figure 1b). Cu2 atoms in the form of [Cu(H_2_O)_2_]^2+^ cations act as diconnecting nodes giving rise altogether to an almost planar hexagonal network (Figure 1). Our studies previously observed these trinuclear units with the mesoxalate ligand.^44,45,47,48,50,52,54^ The mesoxalate ligand loses the two carboxylic and one of the hydroxyl hydrogen atoms and appears as C_3_HO_6_^3–^ (Hmesox)^3–^. Each mesoxalate ligand acts as bidentate with respect to two Cu1 atoms with the alkoxido and the two carboxylate oxygen atoms forming two 5-membered chelate rings (Figure 1b). Moreover, it acts as a bidentate with respect to one Cu2 atom, with two carboxylate oxygens forming a six-membered chelate ring (Figure 1b). Thus, the mesoxalate exhibits a μ_3_-(κO:κO′,κO″:κO″,κO‴′:κO‴″) bridging mode connecting one Cu2 with two Cu1 through two *anti–anti* carboxylate bridges and the two Cu1 atoms through the alkoxido bridge with a Cu1–O3–Cu1^iii^ angle of 124° (Scheme 1c and Figure 1b). This bridging mode is frequently observed in Cu(II)/mesoxalate complexes.^44,45,52^

**Figure 2:**
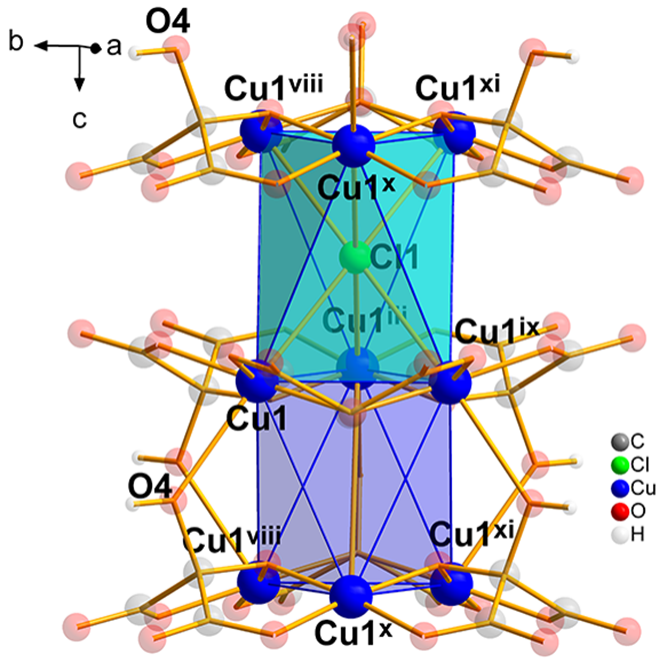
View of the trigonal antiprism metallocages built by the copper(II) ions of three neighboring hexagonal layers in **1**; connected through μ_6_-Cl and mesoxalate oxygen O4. Symmetry codes: (iii) -*y*, *x*-*y*, *z*; (viii) -*x*, -*x*+*y*, -*z*+1; (ix) -*x*+*y*, -*x*, *z*; (x) *y,x,-z;* (xi) *x-y, -y, -z*.

The Cu1 atom exhibits a distorted Jahn–Teller octahedral environment in which the basal positions are filled by mesoxalate-carboxylate (O1) and mesoxalate-alkoxido (O3) oxygens with Cu1–O distances of 1.941 and 1.943 Å, respectively (Figure 1b) (refinement details, main distances, and angles are given in the Supporting Information, Tables S1, S8, and S9). One chloride ion and one alcohol-mesoxalate oxygen, O4^xi^, occupy the two axial positions, at Cu1–Cl1 and Cu1–O4^xi^ distances of 2.920 and 2.673 Å, respectively. The Cl^–^ anion sits in an inversion center and shows a μ_6_-bridging mode linking six Cu1 ions of two trinuclear units of layers a and a′ (Cu1, Cu1^iii^, and Cu1^ix^ with Cu1^viii^, Cu1^x^, Cu^xi^) in a trigonal-antiprismatic anion coordination (Figures 1c and 2, cyan polyhedron). The linking chloride ions alternate the connection with the upper and lower planes, thus producing and stabilizing the three-dimensional network (Figure 1a,c). The shortest interlayer Cu–Cu distance through this bridge is 4.73 Å.

In the opposite side of the connection through the chloride ion, three hydroxyl O4 atoms of three mesoxalate ligands link also between layers at a distance of Cu1–O4 2.673 Å. These three hydroxyl atoms of one trinuclear unit perfectly ensemble in the axial positions of the three Cu1 atoms of the other-layer trinuclear units forming hexanuclear units with Cu(II) ions in the corners of a trigonal antiprism but no chloride ions in the center (Figure 2, dark blue polyhedron). The shortest Cu–Cu separation through this connection is 4.24 Å, somewhat shorter than the one through the μ_6_-Cl link.

The Cu2 atoms display an elongated octahedral environment with four mesoxalate-carboxylate oxygens (O2) of two different trinuclear units in the equatorial positions at a distance of 1.945 Å and two water molecules (O1W) in the long axial positions at a distance of 2.383 Å (Figure 1b).

The anionic network of **1**, [Cu_9_(Hmesox)_6_Cl(H_2_O)_6_]*_n_*^*n*–^, possesses a negative charge, balanced by H_3_O^+^ cations located together with crystallization water molecules in the space between the anionic layers (Figures 1a and S13a). Hydrogen atoms of both hydronium and water molecules could not be found in the structure refinement; thus, both species are indistinguishable.

The anionic network of **2** is identical to that of **1**, and the negative charge is balanced by hydronium and dimethylammonium cations located in the voids together with crystallization water molecules (Figure S13b,c).

### Crystal Structure of **3**

**Figure 3:**
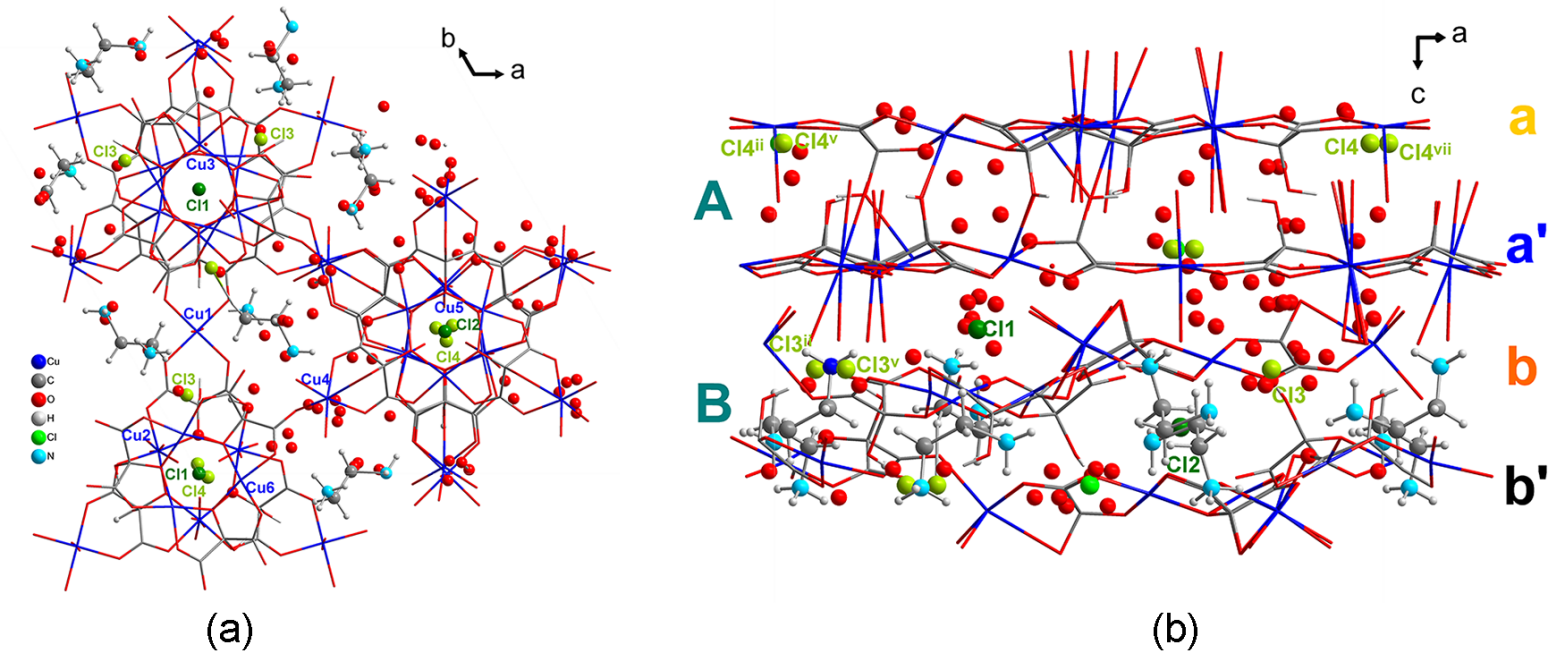
(a) View of a part of the structure of **3** along *c* axis, showing the ethylenediammonium cations, oxygen atoms of hydronium and water molecules occupying the channels along this direction. The anionic nets are represented as ball and sticks. Noncoordinated Cl3 and Cl4 anions are shown as light green balls, and coordinated-to-copper(II) Cl1 and Cl2 anions are shown as dark green balls. (b) View of the structure along the *b* axis. Symmetry codes: (ii) −*x* + *y*, −*x* + 1, *z*; (v) −*y* + 1, *x* – *y* + 1, *z*; (vii) −*x* + *y* + 1, −*x* + 1, *z*.

Compound **3** crystallizes in the centrosymmetric hexagonal *R*3̅ space group (Table S1). The structure consists of a set of connected anionic double layers, which contain the copper atoms, the mesoxalate ligands, as well as some coordination water molecules and chloride anions. The voids are occupied by interstitial water molecules, chloride anions, and disordered ethylenediammonium cations (Figures 3 and S13). There are two crystallographically and chemically different kinds of double layers, designed as “A” and “B”, which stack in an alternate way (ABAB instead of AAAA for **1** and **2**) (Figures 3b and 1c). In turn, each double layer is composed of two single layers related by an inversion center, which we name as “a” and “a′” for A and b and b′ for B. The A–B interlayer connection takes place through large μ6-Cl bridges so that the structure is three-dimensional.

#### A Layer: Copper(II) Ions Cu1, Cu2, and Cu3, Mesoxalate Ligands L_A_ and L_B_

**Figure 4:**
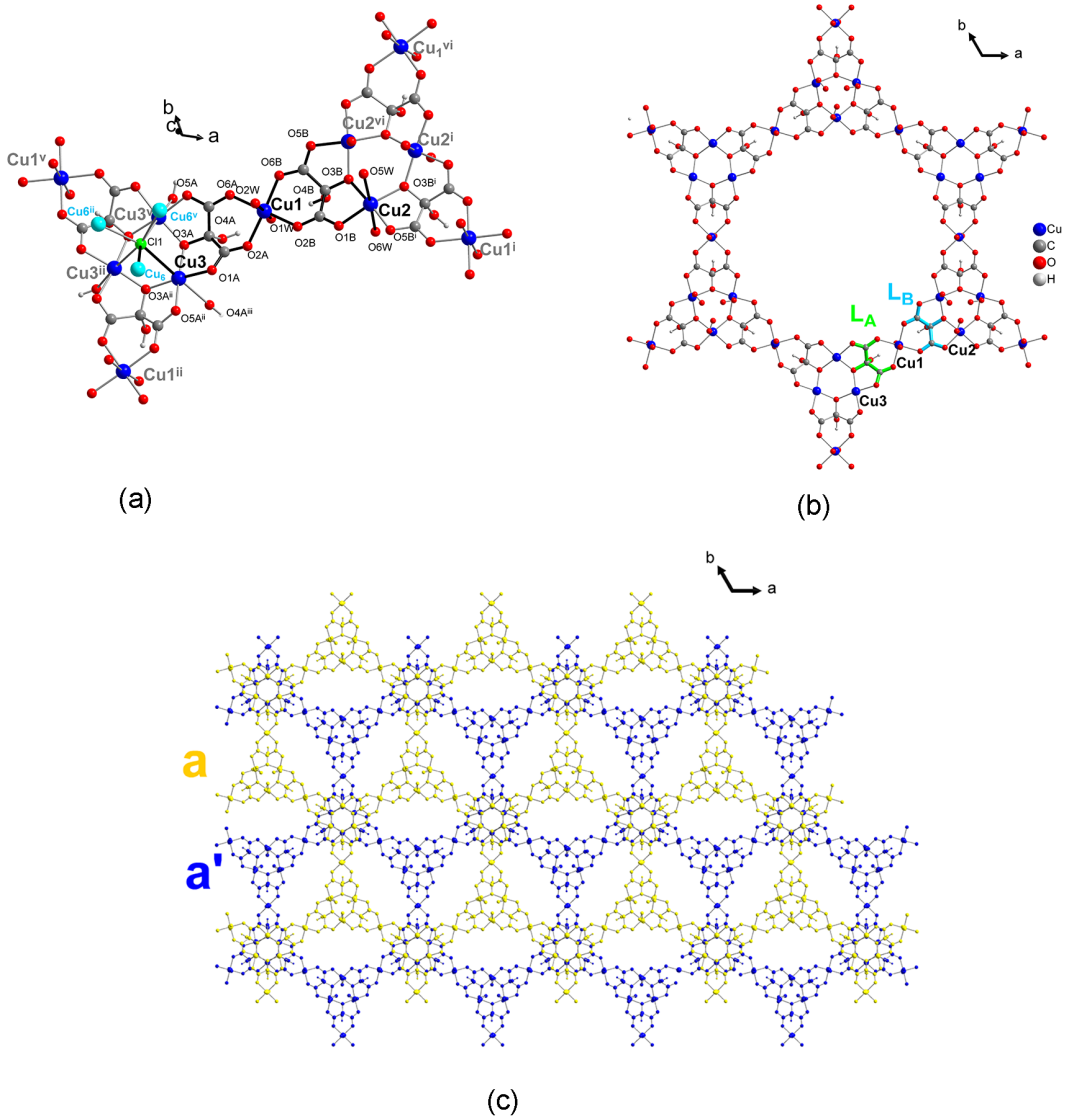
(a) View of the extended asymmetric unit for the A layer. Copper atoms depicted in light blue correspond to copper atoms of the B layer (Cu6). Hydrogen and carbon atoms labels have been omitted for clarity. Bonds depicted in black bind atoms, which are part of the asymmetric unit. (b) View of a hexagonal ring of a single a layer formed by six trinuclear copper(II) entities bridged by six Cu1 atoms. (c) View along the *c* axis of two single a and a′ layers represented in blue and yellow, respectively. Symmetry codes: (i) −*y* + 2, *x* – *y* + 1, *z*; (ii) −*x* + *y*, −*x* + 1, *z*; (iii) *y* – 1/3, −*x* + *y* + 1/3, −*z* + 4/3; (v) −*y* + 1, *x* – *y* + 1, *z*; (vi) −*x* + *y* + 1, −*x* + 2, *z*.

Each layer a or a′ contains three crystallographic independent copper(II) ions, Cu1, Cu2, and Cu3, and two mesoxalate ligands, L_A_ and L_B_ (Figure 4). Cu2 and Cu3, together with mesoxalates L_A_ and L_B_, are involved in the formation of two different trinuclear units, which are bridged by Cu1 atoms to give an almost planar two-dimensional hexagonal network of (6,3) topology (Figure 4a,b). L_A_ and L_B_ mesoxalate ligands appear as C_3_HO_6_^3–^(Hmesox^3–^) and act as tridentate ligands with respect to Cu2 or Cu3 atoms and as bidentate with respect to Cu1, exhibiting the same μ_3_-binding modes as **1–2** (Figure 4a,b and Scheme 1c).

Cu1, Cu2, and Cu3 display a Jahn–Teller elongated octahedral environment (Figure 4a). Four carboxylate oxygen atoms (O2A, O6A and O2B, O6B) from two L_A_ and L_B_ mesoxalate ligands occupy the equatorial positions around Cu1 (distances Cu–O in the range of 1.956–1.992 Å) (Figure 4a). Two coordination water molecules, O1W and O2W, fill the apical positions at 2.328 Å.

Two carboxylate and two alkoxido oxygen atoms from two symmetry-related mesoxalate ligands (O1B, O5B^i^ O3B, and O3B^i^ for Cu2 and O1A, O5A^ii^ and O3A and O3A^ii^ for Cu3) fill the equatorial positions of Cu2 and Cu3. The Cu–O distances span in the range of 1.923–1.960 Å (see distances and angles in Table S11). The two remaining apical positions make the difference between Cu2 and Cu3. In the case of Cu2, two disordered water molecules with partial occupation occupy these positions (O5W and O6W at distances of 2.610 and 2.701 Å), whereas for Cu3, these positions are occupied by an oxygen atom of a hydroxyl group of a mesoxalate from a different a′ layer, O4A^iii^, and a chloride ion, Cl1, at a distance of 2.955 Å (Figure 4a,b).

**Figure 5:**
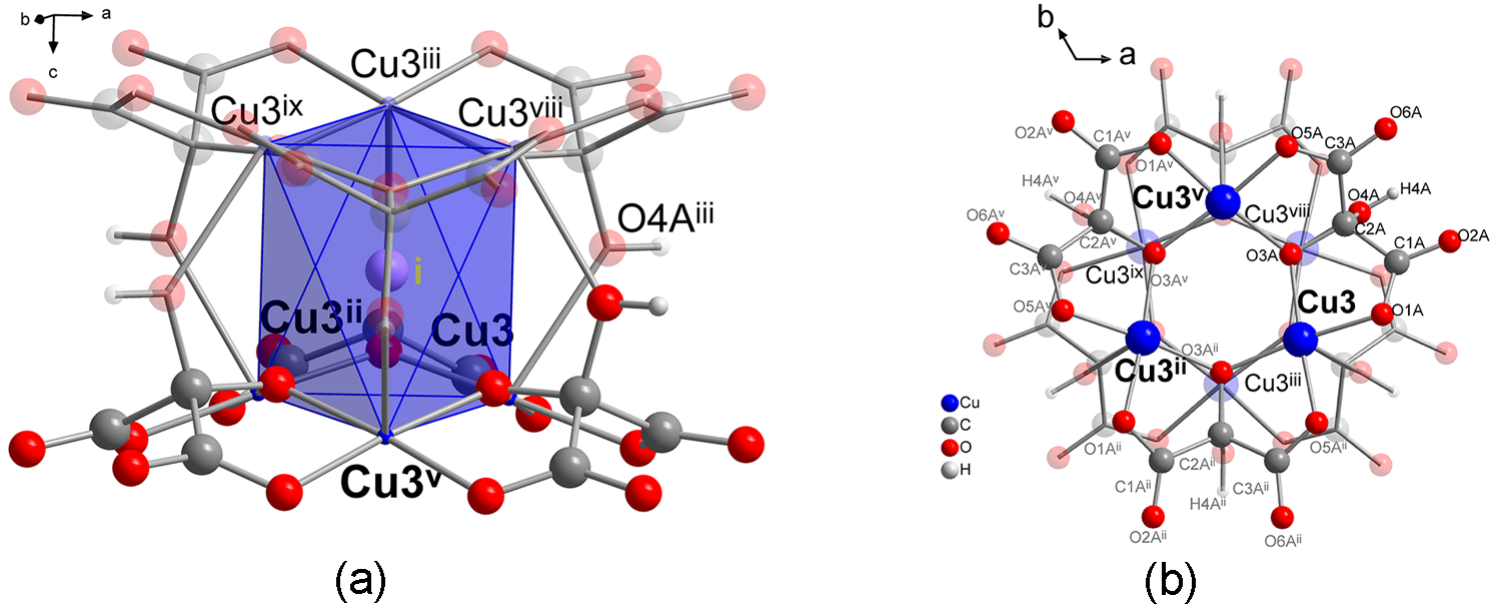
(a) View of the trigonal antiprism displayed by the hexagonal copper(II) entity formed by two trinuclear copper(II) entities of two neighboring layers connected through the mesoxalate hydroxyl oxygen atom O4A; the inversion center, *i*, is also shown. (b) View along the *c* axis of two trinuclear copper(II) entities where the 60° alternated positions for copper(II) atoms of the two trinuclear copper(II) entities can be observed (Cl1 atoms in the apical position to Cu3 connecting to the B layer have been omitted for clarity; labels of C, H, and O atoms of the b′ layer have been also omitted for clarity). Symmetry codes: (ii) −*x* + *y*, −*x* + 1, *z*; (iii) *y* – 1/3, −*x* + *y* + 1/3, −*z* + 4/3; (v) −*y* + 1, *x* – *y* + 1, *z*; (viii) 2/3 + *x* – *y*, 1/3 + *x*, 4/3 – *z;* (ix) 2/3 – *x*, 4/3 *– y*, 4/3 *– z*.

The alcohol bridge through O4A^iii^ atoms connects a layer’s Cu3 atoms and a′, with symmetry-related Cu3 atoms, similarly to that observed for compounds **1** and **2** (Figures 3b, 4a, and 5). The shortest separation between Cu atoms of both layers is 4.351 Å. Crystallization water molecules together with low occupation (0.13) Cl4 chloride ions are located between the a and a′ layers (Figures 3 and S13).

The second apical position of Cu3 is occupied by a chloride atom Cl1, which connects a′ and b layers (Figure 4a).

#### B Layer: Copper(II) Ions Cu4, Cu5, and Cu6, Mesoxalate ligands L_C_ and L_D_

**Figure 6:**
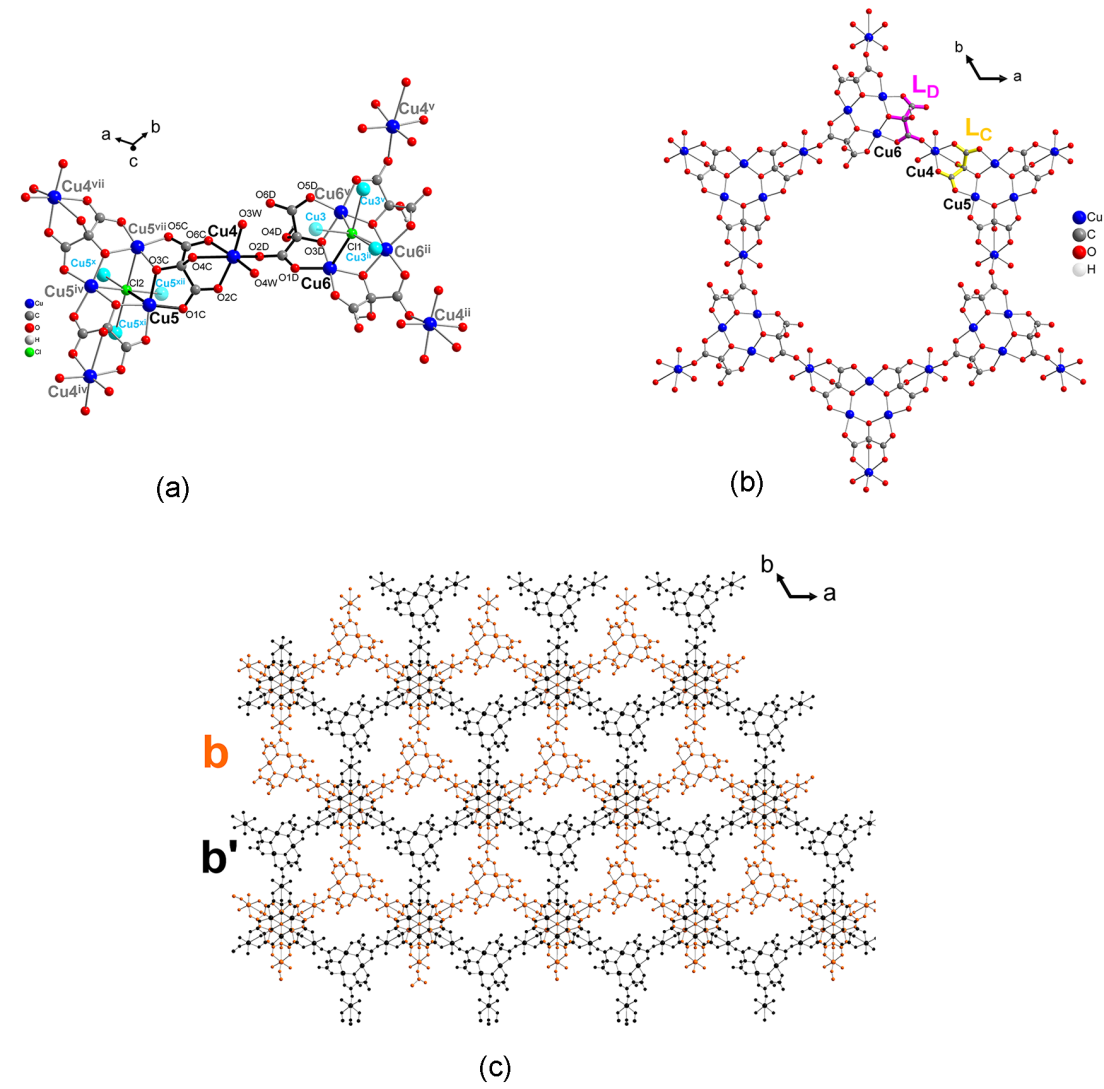
(a) View of the B layer’s extended asymmetric unit. Copper atoms depicted in light blue correspond to either A layer (Cu3) or another B layer (Cu5) copper atoms. Hydrogen and carbon atom labels have been omitted for clarity. Bonds depicted in bold black bind atoms, which are part of the asymmetric unit. (b) View of a hexagonal cycle of a single layer b formed by six trinuclear copper(II) entities. (c) View along the *c* axis of the double-layer B, with the single b and b′ layers represented in orange and black, respectively. Symmetry codes: (ii) −*x* + *y*, −*x* + 1, *z*; (iv) −*y* + 1, *x* – *y*, *z*; (v) −*y* + 1, *x* – *y* + 1, *z*; (vii) −*x* + *y* + 1, −*x* + 1, *z*; (x) 4/3 – *x*, 2/3 *– y*, 5/3 *– z;* (xi) 1/3 + *x* – *y*, −1/3 + *x*, 5/3 – *z*; (xii) 1/3 + *y*, 2/3 – *x* + *y*, 5/3 – *z*.

These layers are formed by two single b and b′ layers (Figure 6a). Every layer contains three crystallographic independent copper(II) ions, Cu3, Cu4, and Cu5 and two crystallographic independent mesoxalate ligands, L_C_ and L_D_ (Figure 6a,b).

Cu5 and Cu6 form two different copper(II) trinuclear units with L_C_ and L_D_ mesoxalate ligands coordinating, respectively, through carboxylate and alkoxido oxygen atoms. Cu4 atoms act as diconnecting nodes bridging the two trinuclear copper(II) units giving rise to a (6,3) network (Figure 6).

L_C_ and L_D_ exhibit different coordination and bridging modes. L_C_ is completely deprotonated C_3_O_6_^4–^(mesox^4–^), which is the second time that it has been reported in the literature^55^ for this ligand and the first time we have observed it within more than 20 compounds with mesoxalate that we have synthesized and results in a shorter C–O distance in this mesoxalate (Figure 6, Scheme 1, Table S2).^44−48,50,51,53,54^

**Scheme 2:**
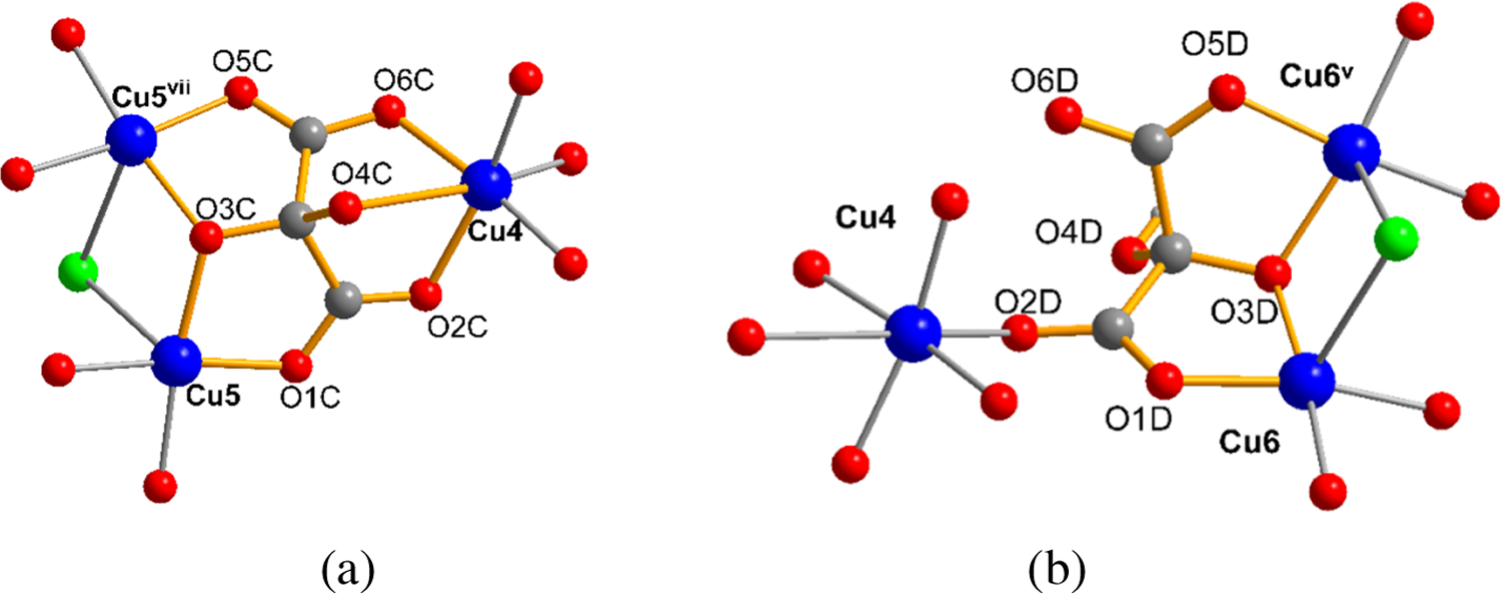
(a) Coordination Mode μ_3_-(κO:κO′; κO″:κO″; κO″:κO‴; κO‴′: κO‴″) for Mesoxalate L_C_. (b) Coordination Mode μ_3_-(κO:κO′,κO″:κO″) Observed for Mesoxalate L_D_ Symmetry codes: (v) −*y* + 1, *x* – *y* + 1, *z*; (vii) −*x* + *y* + 1, −*x* + 1, *z*.

The L_C_ mesoxalate ligand also acts as tridentate with respect to Cu4 and bidentate with respect to Cu5 and Cu5^vii^ with a μ_3_-(κO:κO′; κO″:κO″; κO″:κO‴; κO‴′:κO‴″) binding mode (Scheme 2 and Figure 6a,b). This bridging mode has been found in mesoxalate compounds in which the trinuclear copper(II) entities connect to lanthanoid(III) atoms, but has not been yet observed for transition metal(II) ions connecting to the trinuclear copper(II)-mesoxalate unit.^47,48^

L_D_ mesoxalate in the form C_3_H_1_O_6_^3–^ acts as a bidentate ligand with respect to Cu6 and Cu6^v^ and acts as a monodentate ligand with respect to Cu4; with a μ_3_-(κO:κO′,κO″:κO″) bridging mode (Scheme 2b and Figure 6a). This is the first time that this coordination mode is observed for this ligand.^50^

The connection between two neighboring trinuclear copper(II) units containing Cu5 and Cu6 in the B layer is accomplished by Cu4 atoms. Cu4 connects to Cu5 and Cu5^vii^ through two *anti–anti* carboxylate bridges from mesoxalate L_C_ and to Cu6 by L_D_ through an *anti–syn* carboxylate bridge (Scheme 2).

**Figure 7:**
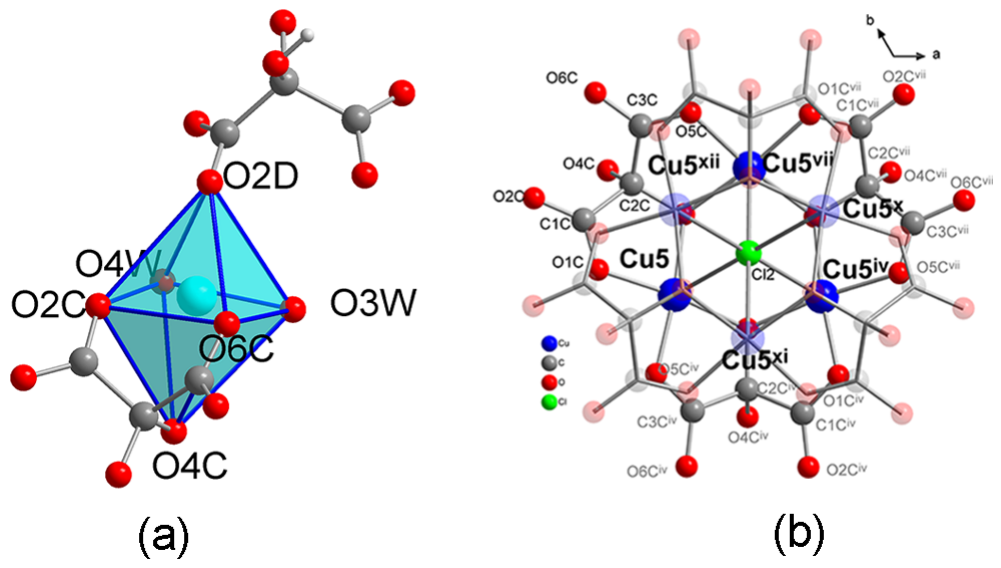
(a) Distorted octahedral coordination environment for Cu4. (b) View along the *c* axis of two trinuclear copper(II) entities containing Cu5 where the 60° alternated positions for copper(II) atoms of the two trinuclear copper(II) entities can be observed (labels of C, H, O atoms of the b′ layer have been omitted for clarity). Symmetry codes: (iv) -*y* + 1, *x* – *y*, *z*; (vii) −*x* + *y* + 1, −*x* + 1, *z*; (x) 4/3 – *x*, 2/3 *– y*, 5/3 *– z*; (xii) 1/3 + *y*, 2/3 – *x* + *y*, 5/3 – *z*.

**Figure 8:**
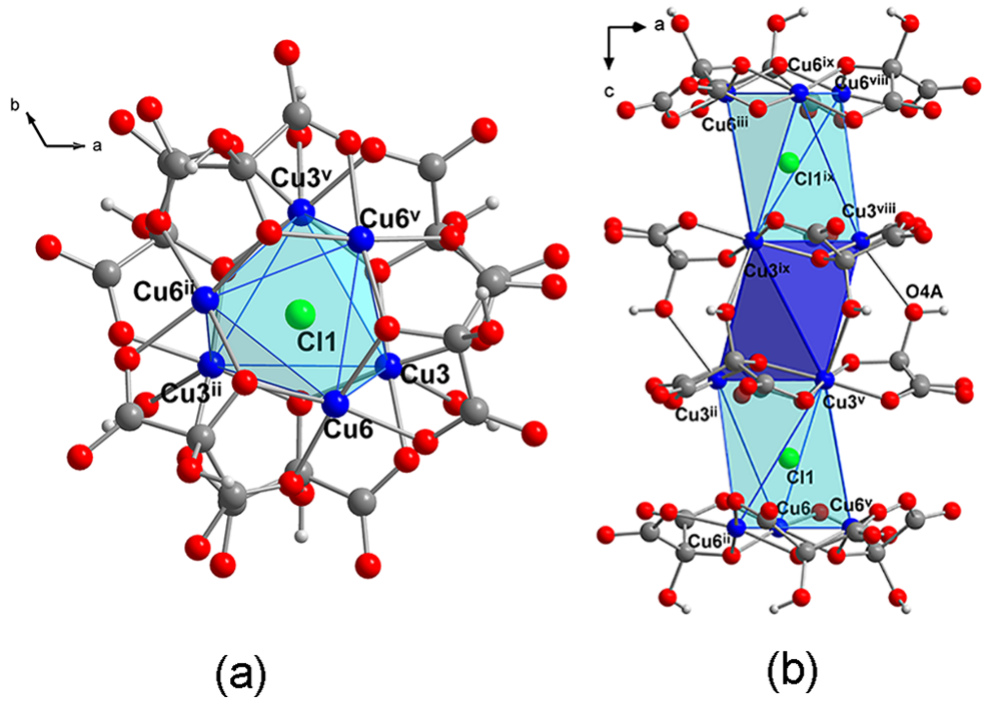
(a) View along the *c* axis of the trinuclear copper(II) units of a and b layers, twisted 38° with respect to each other (b) View along *b* axis of a, a′, b, and b′ layers showing also the polyhedras formed by the two trinuclear copper(II) entities. Hydronium and ethylenediammonium cations, uncoordinated chloride anions (Cl3 and Cl4), and crystallization water molecules between layers have been omitted for clarity. Symmetry codes: (ii) −*x* + *y*, −*x* + 1, *z*; (iii) *y* – 1/3, −*x* + *y* + 1/3, −*z* + 4/3; (v) −*y* + 1, *x* – *y* + 1, *z*; (viii) 2/3 + *x* – *y*, 1/3 + *x*, 4/3 – *z;* (ix) 2/3 – *x*, 4/3 *– y*, 4/3 *– z*.

Cu4 displays a distorted octahedral coordination environment (Figures 7a and 8a) where two of the equatorial positions and one apical position are occupied by two carboxylate oxygens and one alkoxido oxygen of a mesoxalate ligand L_C_ in a *–fac* arrangement, (O2C, O6C, and O4C at distances to Cu4: 1.978, 1.971, and 2.620 Å, respectively). One carboxylate oxygen of ligand L_D_ occupies the remaining apical position (Cu4–O2D distance of 2.262 Å) and two water molecules O3W and O4W occupy the two remaining basal positions, at distances of 1.952 and 1.962 Å.

The coordination environment of Cu5 and Cu6 of the copper(II) ions is similar. Both display a square-planar pyramidal environment with four equatorial positions occupied by two carboxylate oxygen atoms and two alkoxido oxygen atoms from two mesoxalate ligands forming five-membered chelate rings (for Cu5: O1C, O5C^iv^ and O3C, O3C^iv^; for Cu6: O1D, O5D^ii^ and O3D, O3D^ii^) at distances which span the range 1.924–1.970 Å (Figure 6a and Table S11 for distances and angles). The apical position of the Jahn–Teller distorted pyramid is occupied for both Cu5 and Cu6 by two chloride ions; Cl2 and Cl1 respectively, at distances of 2.827 and 2.682 Å.

The connection between b and b′ layers takes place through the Cl2 chloride atom (which sits in an inversion center) occupying the apical position of Cu5 and connecting further to another trinuclear copper(II) entity from b′ containing the atoms (Cu5^x^, Cu5^xi^, Cu5^xii^) (Figures 3b, 6a, and 7b) forming a trigonal antiprism as in the case of the two trinuclear copper(II) units containing Cu3 and Cl1 in *A* layer (Figure 5). These two trinuclear entities, which alternate positions for the copper(II) ions related by the inversion center, are twisted by 60° forming a hexanuclear copper(II) entity (Figure 7b). The inversion center sits on the center of a trigonal antiprism metallocage similar to that formed by Cu3 atoms in the A layer (Figures 3 and 7b), and the shortest separation between Cu atoms of both layers is 4.553 Å. The connection between A and B layers takes place through the Cl1 atom situated in the apical position to Cu3 in layer a′ at a large distance (Cu3–Cl1 = 2.956 Å), which exhibits a μ_6_-bridging mode and connects further to three copper(II) atoms from b layer (Cu6, Cu6^ii^, and Cu6^iii^) at a Cu6–Cl1 distance of 2.69 Å (Figures 4a and 8). The shortest separation between Cu atoms of both a and b layers is 4.310 Å. Bridging chloride ions Cl1, low occupation (0.37) noncoordinated Cl3 anions, and disordered water molecules (Figure 3b) are located between A and B layers. Finally, ethylenediammonium cations can be found in the form enH^+^and enH_2_^+^ and occupy the space between layers b and b′, where Cl2 atoms occur (Figures 3b and S13).

### Magnetic Properties

**Figure 9:**
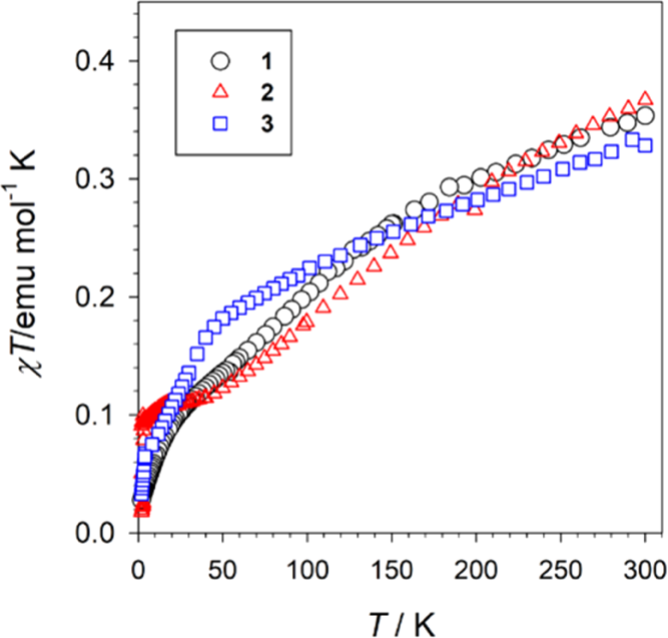
Temperature dependence of the χ*T* product for compounds 1–3. χ refers to the magnetic susceptibility per mole of Cu(II) ion.

The temperature dependence of the χ*T* product for compounds **1–3** is shown in Figure 9, where χ corresponds to the magnetic susceptibility per mole of Cu(II) ion.

The room-temperature χ*T* values range from 0.31 to 0.34 emu mol^–1^ K. Values which are lower than those expected for an uncoupled Cu(II) ion, showing that all of the compounds have some antiferromagnetic coupling even at room temperature (expected 0.41 emu mol^–1^ K, *g* = 2.10 and *S* = 0.5). On lowering *T*, the χ*T* plots continuously decrease, revealing an overall antiferromagnetic interaction for all of the compounds. Nevertheless, there is no long-range antiferromagnetic ordering since the *χ* vs *T* plots do not show any maximum in the whole temperature range (Figure S5). The compounds do not exhibit identical behavior, but the overall behavior is very similar, with a predominance of antiferromagnetic interactions.

**Figure 10:**
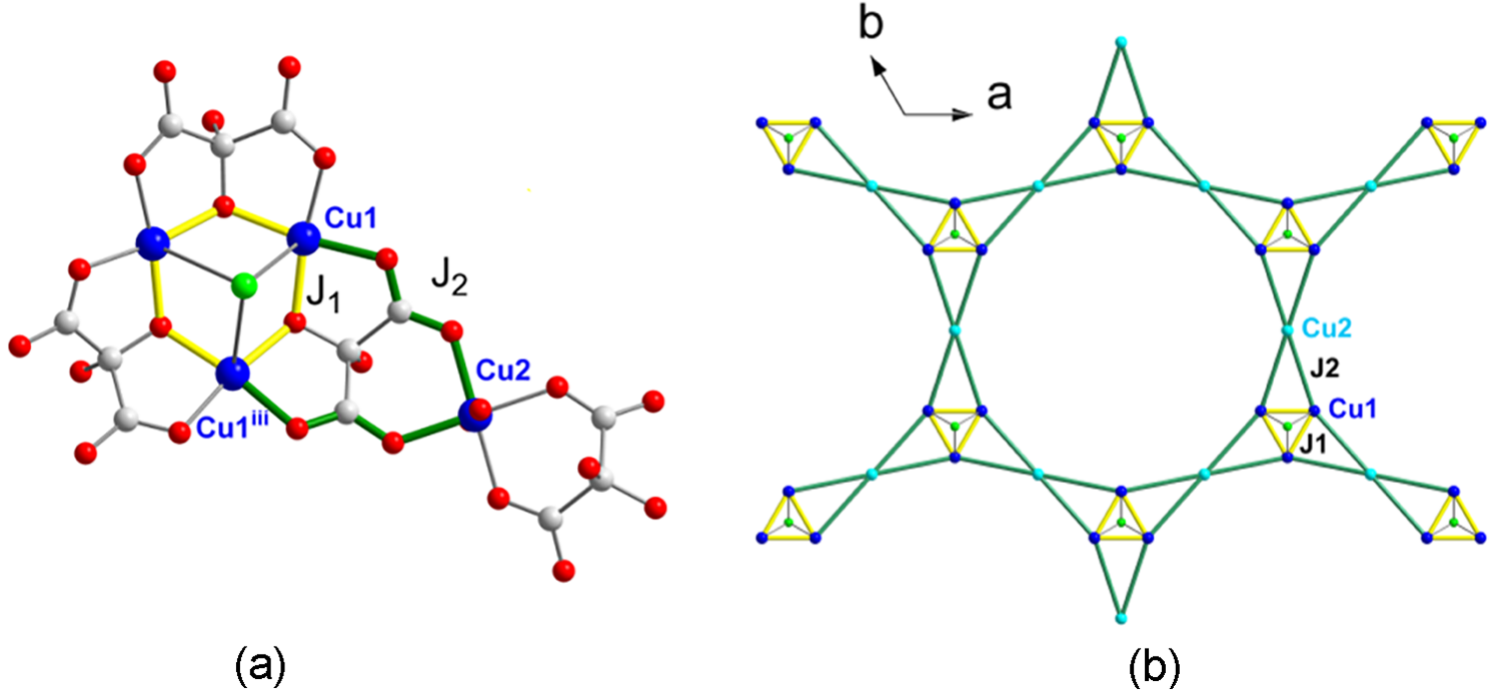
(a) Fraction of the structure of **1**. Three alkoxido-bridged Cu1 ions form an equilateral triangle. Two Cu1 ions of the equilateral triangle are connected through two anti–anti carboxylate bridges to Cu2 atoms, the three atoms forming isosceles triangles. (b) Combination of equilateral (yellow) and isosceles triangles (yellow and green sides) gives the two-dimensional layers in **1** and **2**.

Compounds **1** and **2** show four different magnetic exchange pathways and their corresponding four magnetic coupling constants. One through the alkoxido bridges within the copper(II) triangles, *J*_1_, another one through the *anti–anti* carboxylate bridges, *J*_2_, one-third through the μ_6_-Cl ions, *J*_3_, and finally another one through the alcohol bridges, *J*_4_ (Figures 10, S6, and S7). These last two interactions are not predominant due to the long Cu···Cu distances and the low effectivity of the bridges in the mediation of the magnetic coupling. Focusing on *J*_1_ and *J*_2_, we find alkoxido-bridged copper(II) triangles that are linked through *anti–anti* carboxylate-bridged copper(II) ions to give an alternating equilateral-isosceles double-triangular infinite two-dimensional network (Figure 10b). As far as we know, there is no numerical expression derived from that structure to which the magnetic data could be fitted, and the magnetic coupling constants cannot be calculated this way. Nevertheless, we can anticipate antiferromagnetic interactions for *J*_1_ with Cu1–O3–Cu1^iii^ angles of 124.17(25)° and 124.18(12)° in **1** and **2**, respectively, and also antiferromagnetic coupling for the Cu(II) ions bridged by the *anti–anti* carboxylate groups, *J*_2_.^50,56,57^

Similar, but more complicated is what we find in **3**. In this compound, we have two kinds of layers (A and B) with eight magnetic exchange pathways in total within the two different layers and two additional interlayer couplings to reach a total number of 10 magnetic exchange pathways (Figures S8 and S9). *J*_1_, *J*_4_, *J*_5_, and *J*_8_ refer to magnetic couplings through alkoxido bridges, and *J*_2_, *J*_3_, *J*_6_, and *J*_7_ refer to couplings through carboxylate bridges. *J*_9_ is the coupling among Cu3 ions through the apical μ_6_-Cl bridges, and *J*_10_ is the coupling through the alcohol bridges (Figures 8 and S12). Even discarding interlaminar couplings, there is no mathematical expression for the analysis of the magnetic susceptibility data for this much more complex connectivity and numerical values cannot be given.

Nevertheless, a detailed revision of the structural parameters involved in the different magnetic exchange pathways (Tables S9–S11) points to the predominance of antiferromagnetic couplings among the Cu(II) ions within the layers and almost no interlayer magnetic interactions.

It is known that these antiferromagnetically coupled triangular networks are not expected to show long-range AF ordering within the layers because of the spin frustration, which is in agreement with our observations (Figure S5).^58,59^ On the other hand, they are expected to exhibit exotic phenomena such as spin liquid or spin condensation, but that study is above the scope of this work.^60,61^

### Density Functional Theory (DFT) Calculation of the Magnetic Coupling Constants

Due to the complexity of the **1–3** networks, it is not possible to calculate the values of the magnetic coupling constants by fitting the magnetic susceptibility data to any numerical expression. To calculate those parameters, we performed some DFT calculations according to the descriptions given in the Experimental Section. The values of the energy and spin number of the different spin states calculated for the **1–3** compounds are given in the Supporting Information. We have not calculated all of the magnetic coupling constants of all of the magnetic exchange pathways, but we calculated the values of six magnetic coupling constants corresponding to alkoxide–bridge interactions, three for carboxylate bridges, two for chloride bridges, and one for the alcohol bridge to have a representative sample for each type of magnetic exchange pathway. The values are given in Table 1 together with some related structural parameters or bridging modes.

**Table 1:** Magnetic Coupling Constants and Corresponding Main Structural Parameters

compound	bridge alkoxido	*J* (cm^–1^)	bridge carboxylate	*J* (cm^–1^)	bridge μ_6_-Cl	*J* (cm^–1^)
**1**	Cu1–O2–Cu1^iii^	*J*_1_ = −97	Cu1···Cu2	*J*_2_ = −77	2.9205(9) Å	*J*_3_ = −0.23
	124.2(2)°	*J*_1_ = −108^a^	*anti–anti*			
**2**	Cu1–O2–Cu1^iii^	*J*_1_ = −98			2.9256(1) Å	*J*_3_ = −0.21
	124.2(2)°					
**3**	Cu6–O3D–Cu6^v^	*J*_5_ = −25.1	Cu6···Cu4	*J*_6_ = +38		
	111.89(2)°		*anti–syn*			
**3**	Cu5–O3C–Cu5^iv^	*J*_8_= −6.5	Cu5···Cu4	*J*_7_ = −142		
	118.7(2)°		*anti–anti*			
**3**	Cu3–O3A–Cu3^v^	*J*_1_ = −125			alcohol	*J*_9_ = −0.15
	126°				Cu3(ROH)Cu3	

a Calculation performed with a different set of atoms, see the SI for details.

The values for compounds **1** and **2** are expected to be almost identical due to the almost identical structure of the Cu(II)/mesoxalate network. To check this, we calculated the *J*_1_ and *J*_3_ for fragments of both networks; then, we did not find it necessary to calculate *J*_2_ for **2**. We have calculated *J*_1_ with two different sets of atoms for **1**, and we find a mean value of 103 cm^–1^, which gives a standard deviation of 6 cm^–1^. This value is low and indicates that the method is consistent and that the selected set of atoms has not a critical impact on the value of the magnetic coupling constant.

#### Alkoxido Bridge Interactions

The coupling through the alkoxido bridge in **1**–**3** compounds is antiferromagnetic with values in the range of −6.5 cm^–1^ to −125 cm^–1^. The Cu(II) ions are in a Jahn–Teller elongated octahedral coordination with the alkoxido groups connecting equatorial positions of the Cu(II) ions (Figures 4, 7, and 10). In this geometry, the spin density is located mainly in these equatorial positions (d_*x*^2^–*y*^2^_ orbitals), and the magnetic coupling is highly dependent on the Cu–O–Cu angle and on the hinge distortion.^62−65^ In compounds **1**–**3**, the lower antiferromagnetic values are found for the smaller Cu–O–Cu angles, increasing for larger angles. The values and trend agree with our previous studies in copper(II)/mesoxalate complexes.^44,50^ Nevertheless, *J*_5_ in **3** is somewhat more antiferromagnetic than the expected value; we think that the ferromagnetic interaction with the surrounding Cu4 ions enhances the antiferromagnetic coupling among the Cu6 ions within the triangular unit.

#### Carboxylate Bridge Interactions

In Table 1, we observe a value of *J*_6_ = +37.6 cm^–1^ for the unique *anti–syn* bridging mode in **3** (Figures S9 and S11) and values of *J*_2_ = −77 cm^–1^ and *J*_7_ = −142 cm^–1^ for the *anti–anti* bridging modes in **1** (Figures S6) and **3** (Figure S10), respectively. The antiferromagnetic or ferromagnetic nature of the coupling is explained as a function of the overlap of the orbitals bearing the unpaired electrons with those of the bridge.^57,66−71^ The carboxylate bridge connects equatorial positions of both copper(II) with the maximum overlap for the *anti–anti* configuration, but the coupling through the carboxylate group of the fully deprotonated mesoxalate L_C_ in **3** (*J*_7_ = −142 cm^–1^) is much stronger than that found in **1** (*J*_2_ = −77 cm^–1^). The μ_3_-(κO:κO′,κO″:κO″,κO″:κO″′,κO‴′:κO‴″) bridging mode of this mesoxalate in **3** (Figure S11) decreases the Cu4–Cu5 distance (5.31 Å mean value) compared to that of **1** (Cu1–Cu2, 5.52 Å), explaining the stronger antiferromagnetic interaction through this exchange pathway.

On the other hand, accidental orthogonality occurs for the *anti–syn* bridging mode (Figure 6) that minimizes the antiferromagnetic contribution, becoming dominant the ferromagnetic one.^57,63,68^ This ferromagnetic coupling between Cu4 and Cu6 through the *anti–syn* carboxylate bridge is the most significant difference in the magnetic behavior between **3** and **1**–**2**. Nevertheless, despite its ferromagnetic nature, it does not have any evident impact on the magnetic behavior of the compound since it is surrounded by antiferromagnetic interactions and lacks continuity along the plane.

#### Interlayer Interactions

The magnetic coupling through the μ_6_-Cl and the alcohol groups that interconnect trinuclear units of different layers are very weak (*J*_3_ = −0.1 cm^–1^ and *J*_9_ = −0.15 cm^–1^). Both chloride and alcohol bridges connect axial positions of the Cu(II) ions in which the spin density is very low (d_*z*^2^_ orbitals). Thus, the interlayer interactions are much weaker than those through the alkoxido and carboxylato bridges that occur within the same layer. Therefore, from the point of view of the magnetic interaction, these compounds can be considered as two-dimensional, which could also explain the absence of a long-range magnetic ordering in the low-temperature region.^58,59^

### Conductivity Studies

The presence and the proximity between the lattice water molecules in the crystal structures of these compounds suggest the possibility of proton conduction at low temperatures. The thermogravimetric analysis indicates that water release occurs in N_2_ atmosphere between room temperature and approximately 150 °C for **1–3**. The compounds begin to decompose above this temperature (Figure S2). The estimated water release is comparable for all compounds. Due to the limited thermal stability range of these compounds (Figure S2), the electrical characterization was limited to the temperature range of 25–80 °C.

**Figure 11:**
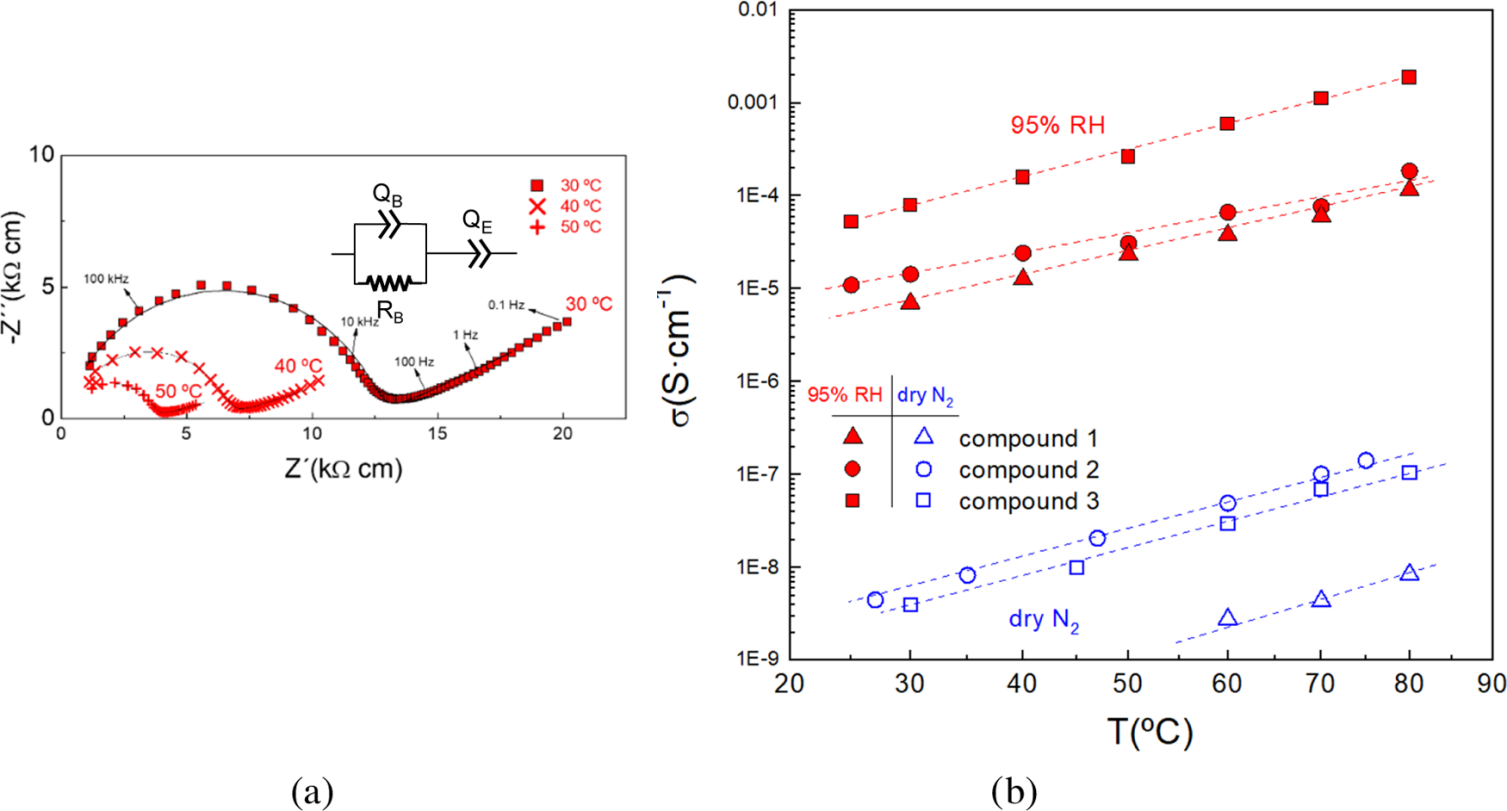
(a) Nyquist plots at different temperatures for compound **2** and the equivalent circuit used to fit the data. (b) Variation of the conductivity with the temperature for the different compounds in wet air (95% RH) and dry N_2_.

The conductivity was determined in both dry and wet atmospheres to elucidate the conducting characteristic of the materials. Representative impedance spectra for **2** at different temperatures in 95% RH are shown in Figure 11. The spectra are formed by a depressed semicircle, attributed to the electrolyte conduction, followed by a spike at a low frequency associated with the electrode polarization at the electrode/electrolyte interface. This last process indicates a partial-blocking electrode response consistent with ionic migration.

The spectra were analyzed by the equivalent circuit displayed in the inset of Figure 11a, where an RQ element (a resistance in parallel with a constant phase element) and a serial Q element are used to simulate the electrolyte conduction and the electrode response, respectively. Note that a constant phase element is applied instead of a simple capacitor to consider the nonhomogeneous system. The capacitance of the semicircle is about 15 pF cm^–1^, and therefore, is unequivocally assigned to bulk conduction, indicating that grain boundary contribution related to internal interfaces and porosity is negligible.

The conductivity was obtained from the resistance *R*, determined by the equivalent circuit fitting, and the geometric factor of the pellets. The total conductivity as a function of the temperature during the cooling process is shown in Figure 11b.

For all of the compounds, it is observed that the conductivity increases with temperature, both in dry N_2_ and at 95% HR due to the higher mobility of the carriers (water molecules and cations) on increasing the temperature (Table 2), suggesting a predominant Grothuss mechanism.^72^

**Table 2:** Conductivity Values for Compounds **1–3** Measured at Low and High Temperatures in 95% Relative Humidity and in Dry Nitrogen

	σ (S cm^–1^) at low temperature^a^		σ (S cm^–1^) at high temperature^a^	
compound	95% RH	dry N_2_	95% RH	dry N_2_
1	d7.05 × 10^–6^	b	f1.16 × 10^–4^	f8.49 × 10^–9^
2	b1.1 × 10^–5^	c4.5 × 10^–9^	f1.85 × 10^–4^	e1.43 × 10^–7^
3	b5.26 × 10^–5^	d4 × 10^–9^	f1.87 × 10^–3^	f1.05 × 10^–7^

a *T* stabilized value of the measurements.

b 25 °C.

c 27 °C.

d 30 °C.

e 75 °C.

f 80 °C.

This is in contrast to our previous studies where we observed a drop in conductivity on increasing temperature, which we associated with dehydration of the material.^49^ In comparison, the compounds studied herein lose their water molecules of crystallization at higher temperatures than those previously studied (see Figure S2) due to stronger framework–crystallization water interactions, making them suitable to display higher proton conductivities at elevated temperatures.

Comparing the values of dry N_2_ for all of the compounds, it can be noted that the dehydration under these conditions leads to very low values independently of the temperature; this is more obvious for **1** at 25 °C, whose conductivity values were not determined at a low temperature due to the high electrical resistance of the pellets, above the detection limit of the equipment.

Compound **3** exhibits the highest conductivity in the whole temperature range studied, i.e., 1.87 × 10^–3^ S cm^–1^ at 80 °C and 95% RH compared to 1.16 × 10^–4^ S cm^–1^ for **1** (Table 2). This conductivity value for **3** at 80 °C and 95% RH is among the highest reported for aliphatic carboxylate-based proton conductive MOFs under the same conditions of humidity and temperature.^73,74^ The corresponding activation energies are in the range of 0.43–0.5 eV, typical of predominant Grotthuss conduction mechanism through water molecules interconnected via H*-*bonds.^72^ Nevertheless, the slightly high values of activation energies, compared to the theoretical value of 0.4 eV, might also suggest the presence of direct diffusion of protons through the vehicle mechanism.^23,29,43,75^ In fact, the refinement of the structures through single-crystal X-ray diffraction for compounds **1–3** shows several oxygen atom sites with partial occupation, as well as partially occupied nitrogen atom sites for samples **2** and **3**, which could be an evidence of direct jump diffusion.^11,12^

It is worth noting the behavior of the compounds under humid conditions. At 80 °C, the conductivity of compound **3** is 1 order of magnitude higher than that displayed by **1** and **2**, and this difference could be explained attending to a combination of several factors. First, compound **3** displays an extra negative charge in the network originated by the complete deprotonation of the mesoxalate L_C_; consequently, an extra proton carrier is needed in its structure to balance the negative charge, namely, ethylenediammonium cations (Figures 3, 6, and S13). If we calculate the number of proton carriers for **1–3**, we observe that in fact, **3** displays 2 × 10^27^ proton carriers/m^3^, which is a value 3 times higher than the ones displayed by compounds **1** and **2**, 9.4 × 10^26^ and 9.3 × 10^26^ carriers/m^3^, respectively. The number of carriers for **1** and **2** is comparable—hydronium cations for **1** and hydronium and methylammonium cations for **2**—with the same proportion in the formula of the compounds (Figures 1 and S13). We argue that the similar number of proton carriers is the reason of the similar conductivity values, which they display under humid conditions.

Another factor that could explain the higher conductivity of **3** is the number of coordinated water molecules, which are more acidic than free water molecules,^35,36^ favoring the proton transfer along the structure. Compound **3** displays a higher number of coordination water molecules, 8.56 atom %, while **1** and **2** display 6.51 and 6.43 atom % respectively (Figures 3 and S13).

The conductivity decreases almost 4 orders of magnitude in dry N_2_. This behavior is clearly related to the release of water molecules, disrupting the proton movement path, and consequently the proton conductivity.

After the electrochemical characterization in humidified atmosphere, the samples were analyzed by X-ray powder diffraction, and we could observe that they have maintained the integrity of the structure (Figure S4).

## Conclusions

Three new copper(II)-mesoxalate compounds have been prepared. All of the compounds constructed by pillaring low-dimensional hexagonal (6,3) networks connected through mesoxalate and chloride bridges display a three-dimensional anionic network with the same Cu(II)/mesoxalate ratio of 1.5:1. Crystallization water molecules and counterions are located in the interlayer space. The mesoxalate ligand and the copper(II) ions are selected to provide magnetic properties to the material, whereas the counterions located in the interlaminar space (H_3_O^+^, NH_2_Me_2_^+^, enH_2_^2+^, enH^+^, and Cl^–^) provide proton conductivity. The strategy followed in this work leads to paramagnetic proton-conducting materials.

The paramagnetic copper(II) ions connected through mesoxalate-alkoxido and mesoxalate-carboxylate bridges lead to a predominant antiferromagnetic coupling in all of the compounds. The carboxylate bridge interactions are mostly antiferromagnetic and strong, which reinforces the antiferromagnetic character of the material. Although the interaction through the *anti–syn* pathway is ferromagnetic, it has little presence and no impact on the material’s properties. DFT calculations confirm the interactions’ predominant antiferromagnetic nature.

The counterions provide the necessary positive charge to neutralize the anionic network and condition the structure of the compounds resulting in a lower symmetry in **3**. We have been able to improve the conductivity values by the selection of the counterions, obtaining that compound **3** exhibits conductivity values 1 order of magnitude higher than compounds **1** and **2**, due mainly to the higher number of proton carriers and the higher number of acidic coordination water molecules. The conductivity value for **3** at 80 °C and 95% relative humidity is 1.87 × 10^–3^ S cm^–1^ and is the highest reported for aliphatic carboxylate-based proton conductive MOFs under the same conditions of humidity and temperature.

## Experimental Section

### Materials and Methods

Reagents were obtained from commercial sources and used without further purification. Elemental analyses for **1** were performed with an EA1108 CHNS-O microanalytical Analyzer, with a PerkinElmer CHN 2400 Analyzer for **2** and with a CHNS TruSpec Micro LECO Analyzer for **3**. IR spectra (400–4000 cm^–1^) were recorded on a Thermo Nicolet Avatar 360 FT-IR spectrometer with the sample prepared as KBr disks. Thermogravimetric and differential thermal analysis (TG-DTA) curves for **1** and **2** were recorded in an SDT-Q600 from TA Instruments at a heating/cooling rate of 5 °C·min^–1^ under N_2_ from room temperature (RT) to 1000 °C. For **3**, thermogravimetric analysis (TGA) was done with a Netzsch TG209 F3 Tarsus (Netzsch, Selb, Germany) in the range of 20–600 °C with a heating range of 5 °C min^–1^ under a nitrogen atmosphere. X-ray powder diffraction patterns on the polycrystalline samples of **1–3** were collected with a PANalytical X’Pert X-ray diffractometer (Cu K_α,1_ radiation = 1.54184 Å) at room temperature.

### Syntheses of **1**–**3**

(H_3_O)[Cu_9_(Hmesox)_6_(H_2_O)_6_Cl]·8H_2_O **(1)**,

(NH_2_Me_2_)_0.4_(H_3_O)_0.6_[Cu_9_(Hmesox)_6_(H_2_O)_6_Cl]·8 H_2_O**(2)**

(enH_2_)_0.25_(enH)_1.5_[Cu_6_(Hmesox)_3_(mesox)(H_2_O)_6_Cl_0.5_]·Cl_0.5_·5.25H_2_O **(3)**

The formula of the compounds was elucidated through elemental analyses, but the water content was estimated through thermogravimetric analyses since the samples used to measure the elemental analyses were dried in an oven at 30 °C for 2 days.

Compounds **1–3** were prepared following a similar procedure. First, a mesoxalic acid solution was obtained by adding a cationic exchange resin (Amberlite IR-120, 5 g) to a suspension of mesoxalic acid disodium salt (540 mg, 3 mmol) in 27 mL of water. Afterward, this solution was slowly added through a Buchner funnel to copper(II) basic carbonate (330 mg, 3 mmol) placed in a round-bottom flask. The reaction was performed for 20 minutes at 30 °C under stirring and soft vacuum to remove the CO_2_ evolved. Initially, the solution was dark green, but at the end of the reaction, it became blue, indicating the presence of the copper(II) trinuclear species, H_3_[Cu_3_(Hmesox)_3_]. Then, 27 mL of H_3_[Cu_3_(Hmesox)_3_] precursor solution (pH = 2.75) was divided into three aliquots of 9 mL each. Solid copper(II) chloride tetrahydrate (342 mg, 2 mmol) was added to each aliquot under stirring.

After that, each aliquote was added to the following solutions drop by drop until the pH of the solution reached 3.0: 1 M tetrabutylammonium hydroxide,1.5 M dimethylamine, and 1.5 M ethylenediamine for **1–3**, respectively.

The solutions were allowed to crystallize at 23 °C in an oven, and after a few days, rhombic blue crystals were formed by **1** and **2**, while **3** yielded blue twinned elongated rhombic dodecahedral crystals. Yields: 130 mg 48%, 180 mg 67%, and 210 mg 58% and for compounds **1–3**, respectively.

The infrared spectra for compounds **1–3** are shown in Figure S1. For all of the compounds, the most intense bands correspond to asymmetric and symmetric carboxylate absorptions, 1650–1570 cm^–1^ (s,b), 1460–1480 cm^–1^ (s), which overlap with those of N–H (b) in the case of **2–3**. The C–O stretching bands can be found around 1120 cm^–1^, which also overlap with the C–N (s) for **2–3**. O-H (s) bands appear in the range of 3500–3600 and also overlap with the N–H (s) for **2–3**.

Elemental analysis was calculated for: (**1**) C_18_H_33_Cl_1_Cu_9_O_48_ (812.2): C, 13.30; H, 2.03. Found: C, 13.31; H, 1.96. (**2**) C_18.8_H_23_Cl_1_Cu_9_O_42_N_0.4_ (1533.65): C, 14.71; H, 1.50; N, 0.37. Found: C, 14.59; H, 1.56; N, 0.39. (**3**) C_15.5_H_32_Cl_1_Cu_6_N_3.5_O_30.5_ (1171.8): C, 15,857; H, 2.73; N, 4.18. Found: C, 15,62; H, 2.73; N, 4.12.

Despite the formula of all of the compounds being established by elemental analysis, the crystallization water molecules of each compound were estimated taking into account the results of the thermogravimetric analysis, for which the samples were freshly prepared.

### X-ray Crystallography

Suitable single crystals were selected under a polarizing microscope, taken directly from the mother liquors, and covered in protective oil before they were put on a 0.05 mm loop. Single-crystal XRD data for **1** and **2** were collected with an Agilent SuperNova diffractometer with a micro-focus X-ray under Cu Kα radiation (λ = 1.5418 Å) for **1** and Mo Kα (λ = 0.71073 Å) for **2**. CrysalisPro software was used to collect, index, scale, and apply analytical absorption correction based on the multiscan method.^76^ Single-crystal XRD data for **3** were collected with a Bruker Kappa APEX2 CCDX-ray diffractometer with micro-focus tube, Cu Kα radiation (λ = 1.54184 Å), multilayer mirror system, ω-scans; data collection with APEX2,^77^ cell refinement with SMART and data reduction with SAINT,^77^ experimental absorption correction with SADABS.^78^

#### Structure Analysis and Refinement

All of the structures were solved by direct methods using SHELXL2016,^79^ and refinement was done by full-matrix least-squares on *F*^2^ using the SHELXL2016 program suite and the graphical user interface (GUI) ShelXle^80^ was used. Crystal data and details on the structure refinement are given in Table S1. Graphics were drawn with Diamond.^81^ The simulated PXRD patterns were obtained with Diamond.^81^ Crystallographic data (excluding structure factors) for the structures in this paper have been deposited in the Cambridge Crystallographic Database (CCDC identification numbers can be found in Table S1).

For **1**, hydrogen atoms on the mesoxalate hydroxyl groups were positioned geometrically (O–H = 0.83 Å) and refined using a riding model (AFIX 83) with *U*_iso_(H) = 1.5 *U*_eq_(O). Hydrogen atoms of O1W were found, and the O–H distance was refined with a DFIX command. Oxygen atoms of the hydronium cation and water solvent molecules were located disordered in the voids and PART commands were used, but their hydrogen atoms could not be located. Nevertheless, they have been included in the unit card for the calculation of the sum formula due to their large number. For **2**, hydrogen atoms on mesoxalate hydroxyl groups were positioned geometrically (O–H = 0.83 Å) and refined using a riding model (AFIX 83) with *U*_iso_(H) = 1.5 *U*_eq_(O). The hydrogen H1W atom of O1W—coordination water molecule—was refined using DFIX commands and also were dimethylammonium N1D, C1D, and C2D atoms. PART commands were also used for O2W and N1D, C1D, C2D. Hydrogen atoms of a water molecule of crystallization, O2W, and for dimethylammonium could not be found, but they have been included in the unit card for the calculation of the sum formula.

For **3**, despite the excellent quality of the crystals, the first attempts for solving the structure yielded an incomplete model with spurious peaks in the difference map in nonreasonable positions. This was considered as a symptom of possible twinning and, in fact, considering the existence of a rhombohedral (obverse/reverse) twinning^82^ (twin law 0 1 0 1 0 0 0 0 −1), with a 0.59/0.41 contribution for the obverse/reverse components of the twin, let us find and refine a satisfactory model for this compound. Non-H atoms were refined anisotropically introducing ISOR restrains for copper atoms Cu1, Cu2, and Cu3 and some atoms of ligand L_A_ to avoid unreasonable anisotropic thermal parameters. The water molecules coordinated to Cu2 were found to be disordered between two positions. Peaks in interstitial positions were interpreted according to their environments and their intensities as chloride anions or water molecules, with partial occupancy for chlorine atoms Cl3 and Cl4 and disorder/partial occupancy for some of the water molecules. Hydrogen atoms on mesoxalate hydroxyl groups were positioned geometrically (O–H = 0.83 Å) and refined using a riding model (AFIX 83) with *U*_iso_(H) = 1.5 *U*_eq_(O). Hydrogen atoms of the ethylenediammonium cations were placed in ideal positions and refined in a riding model (secondary hydrogen atoms with AFIX 23 and *U*_iso_(H) = 1.2 *U*_eq_(N), and quaternary hydrogen atoms with AFIX 33 and *U*_iso_(H) = 1.2 *U*_eq_(N). Elemental analysis data indicate the presence of a proportion of ethylenediammonium cations higher (around 0.75 mole per mole of compound) than that included in the crystallographic model; we think this moiety may be placed in voids of the structure in a disordered way, being indistinguishable from the interstitial disordered water molecules and, hence, we have not been able to introduce it in our model in a reliable way.

Hydrogen atoms of the water solvent molecules were not located since related oxygen atoms were heavily disordered in the voids, but they have been included in the unit card.

### Magnetic Measurements

Magnetic susceptibility measurements on polycrystalline samples were carried out by means of a Quantum Design SQUID MPMS XL magnetometer. The direct current DC measurements were performed in the temperature range of 1.9–300 K at applied magnetic fields of 1000 Oe for *T* < 15 K, and 10 000 Oe for *T* above 15 K. Diamagnetic corrections of the constituent atoms were estimated from Pascal’s constants,^63^ and experimental susceptibilities were also corrected for the temperature-independent paramagnetism and the magnetization of the sample holder.

### DFT Calculations

Broken-symmetry spin-unrestricted DFT calculations were performed at the B3LYP level through the Gaussian09 suite of programs with a triple-ζ (TZV) basis set for all of the atoms.^83−90^ All calculations were performed with the atomic positions taken from the cif files of compounds **1–3**, without structural optimization. Alcohol and water molecules’ hydrogen atoms were added geometrically. We have carried out the calculations in fragments of the structures of the compounds taking groups of six Cu(II) atoms, including all of the ligands present in their coordination sphere. We have considered that six atoms of Cu(II) are the appropriate number since it allows us to simultaneously calculate the magnetic coupling constants among the Cu(II) ions inside the trinuclear entities, the coupling with those outsides, and also between two trinuclear entities. The multiplicity of each Cu(II) ion is introduced directly in the input file. A calculation with a quadratic convergence criterion is done prior to the self-consistent field calculation with a Conver = 7 convergence criterion.^91,92^ The spin states and the equations to calculate the values of the magnetic coupling constants are given in the SI.

### Proton Conductivity Measurements

The electrical characterization of the materials was performed using cylindrical pellets of 5 and 1 mm diameter and thickness, respectively, which were prepared by pressing approximately 0.2 g of milled crystals in a hydraulic press at 200 MPa. Ag ink (Merck) was coated on both sides of the pellets as the current collector. Impedance spectra were collected using a Solartron 1260 frequency response analyzer in the range of 0.1 Hz to 1 MHz with an applied dc voltage of 0.1 V in both humidified and dry atmospheres. The samples were mounted on an electrochemical cell with Pt meshes, which was introduced in a temperature humidity-controlled chamber (Espec SH- 222) and slowly heated at 0.2 °C min^–1^ up to 85 °C in air atmosphere with 95% relative humidity. Impedance spectra were recorded on cooling in intervals of 30 min over a period of 5 h to ensure thermal stabilization at each temperature. Water condensation on the sample was avoided by reducing first the relative humidity before decreasing the temperature. The spectra were also measured in dry nitrogen (1 ppm H_2_O) to further confirm the proton conduction in the materials. The impedance spectra were analyzed with ZView software (Scribner Associates).

After the electrochemical characterization in humidified atmosphere, the samples were analyzed by X-ray powder diffraction (Figure S4).

## Supporting Information Available

X-ray powder diffractograms, IR spectra, thermogravimetric analysis, single-crystal structure refinement details, magnetic susceptibility plot, DFT calculations details, and main distances and angles (PDF)
