# Supplementary material for: Improvement of the Proton Conduction of Copper(II)-Mesoxalate Metal–Organic Frameworks by Strategic Selection of the Counterions
Source: Inorg Chem. 2022 Jul 15;61(30):11651–66. doi: 10.1021/acs.inorgchem.2c01241 (PMC9377511; doi:10.1021/acs.inorgchem.2c01241)
Supplement: Supplementary file 1 — ic2c01241_si_001.pdf [file ic2c01241_si_001.pdf]

Supporting Information

Improvement of the proton conduction of copper(II)-mesoxalate metal-organic frameworks by strategic selection of the counterions

Beatriz Gil-Hernández,[a,b] Simon Millan ,[c] Irina Gruber,[c] Miguel Quirós,[d] David Marrero-López,[e] Christoph Janiak,[c] and Joaquín Sanchiz*[a,b]*

[a]Departamento de Química, Facultad de Ciencias, Sección Química, Universidad de La Laguna, 38206 La Laguna, Tenerife, Spain

[b]Institute of Materials and Nanotechnology, Universidad de La Laguna, P.O. Box 456, La Laguna, E-38200, Tenerife, Spain

[c]Institut für Anorganische Chemie und Strukturchemie, Heinrich-Heine Universität Düsseldorf, 40204 Düsseldorf, Germany

[d]Departamento de Química Inorgánica, Facultad de Ciencias, Universidad de Granada, 18071 Granada, Spain

[e]Departamento de Física Aplicada I, Campus Teatinos s/n, Universidad de Málaga, 29071 Málaga, Spain

*Corresponding authors. E-mail: beagher@ull.edu.es; jsanchiz@ull.edu.es

Content:

Section 1. Characterization

Section 2. Single-Crystal X-Ray data collection and structure refinement

Section 3. Magnetic Properties

Section 4. DFT calculations

Section 5. Proton conduction

Section 6. Main Distances and Angles for 1-3.

Section 1. Characterization

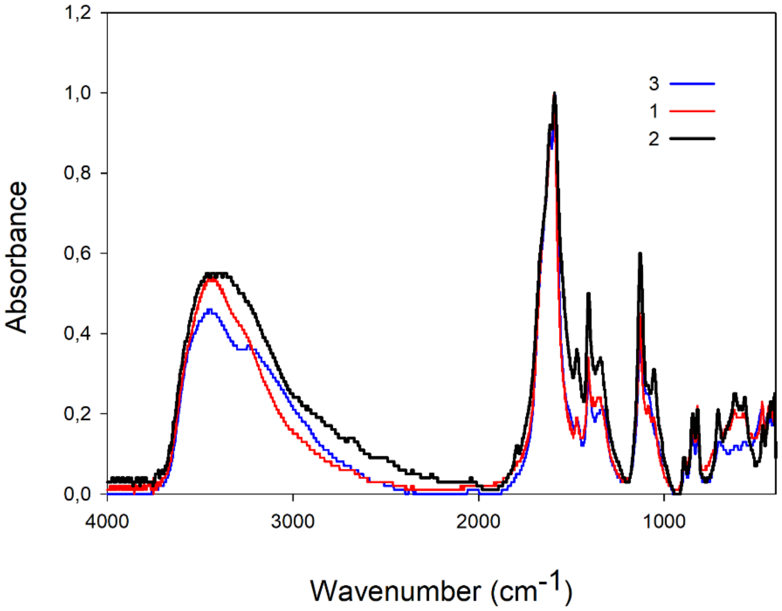

Figure S1. IR spectra for compounds **1-3**.

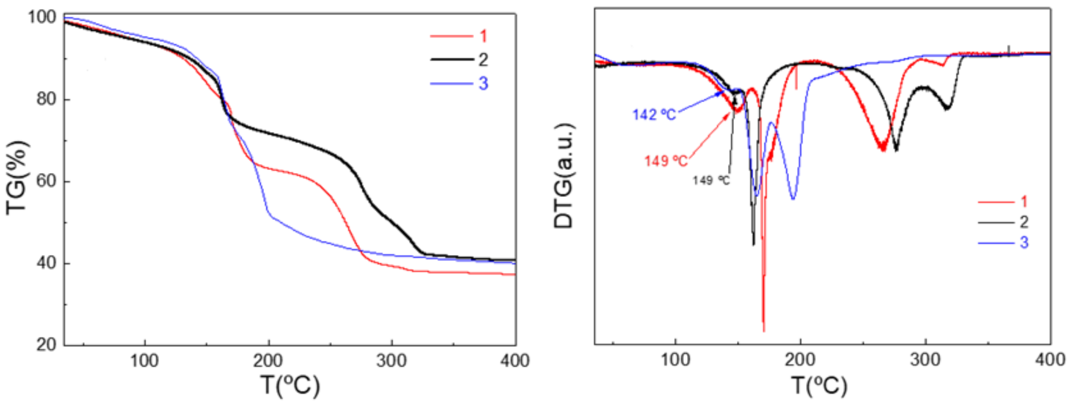

a) b)

Figure S2. Thermogravimetric analysis TGA (a) and derivative thermogravimetry (DTG) (b) for compounds **1-3**.

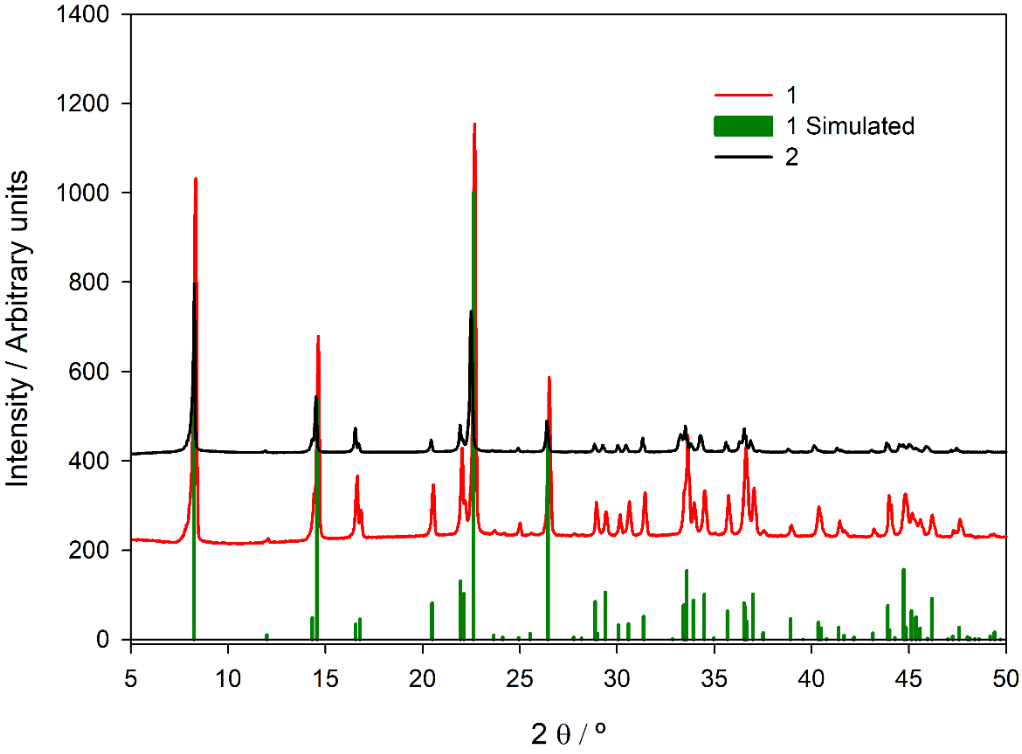

a)

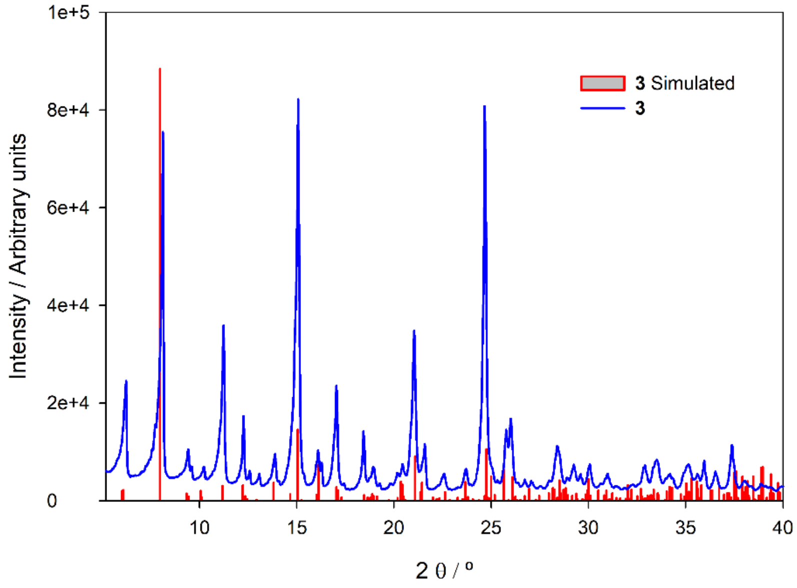

b)

Figure S3. a) X-Ray powder pattern diffractogram of **1**-**2** showing that they are isostructural. In red colour is represented the simulation of the powder diffractogram obtained by single-crystal data of **1**. b) X-Ray powder pattern diffractogram for **3** and the simulation of its powder diffractogram obtained by single-crystal data of **3**.

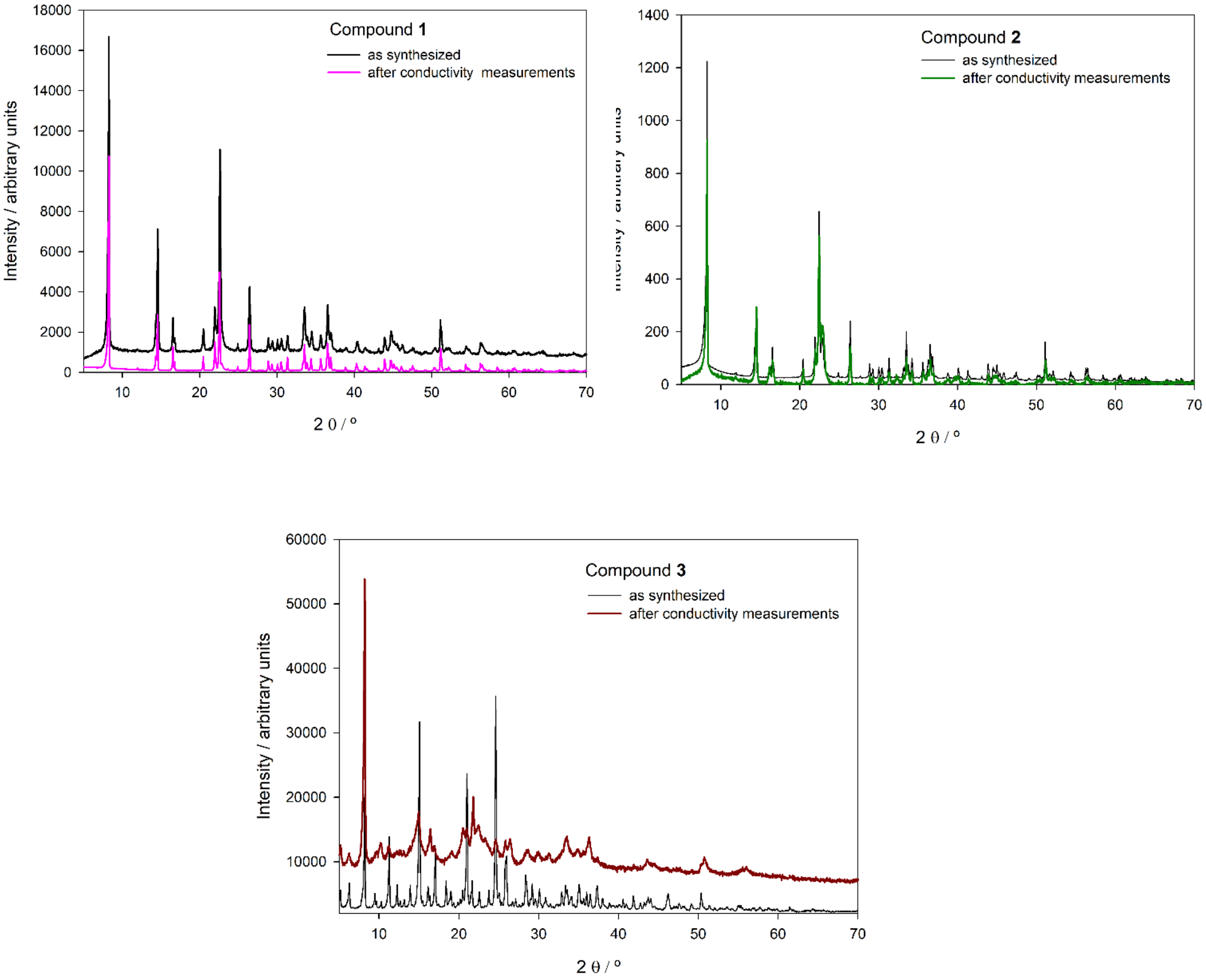

Figure S4: X-Ray powder pattern diffractograms for **1-3** of the as-synthesized compounds and after the conductivity measurements.

Section 2. Single-Crystal structure refinement details

Suitable crystals were carefully selected under a polarizing microscope, covered in protective oil and mounted on a 0.05 mm cryo-loop.

Structure data and refinement:

Table S1. Crystal data and structure refinement details of compounds **1-3**.

Compound	**1**	**2**	**3**
CCDC Number	2049703	2049702	2049704
Empirical formula[a]	$C_{18}H_{37}ClCu_9O_{51}$	$C_{22}H_{37}ClCu_9N_2O_{44}$	$C_{14}H_{38}ClCu_6N_2O_{37}$
M/gmol^{-1}	1676.78	1640.84	1243.15
Temperature (K)	293	293	100
λ/Å	1.54184	0.71073	1.54184
Crystal system, space group	Trigonal, *R*-3*m*	Trigonal, *R*-3*m*	Trigonal, *R*-3
a, *c*(Å)	21.4078 (8), 8.0385 (6)	21.4168 (6), 8.0950 (3)	21.767 (5), 42.878 (10)
V (Å^3)	3190.4 (3)	3215.6 (2)	17595 (8)
Z	3	3	18
D_{calc}/gcm^{-3}	2.618	2.542	2.112
μ / (mm^{-1})	6.83	4.58	5.31
Theta range/°	4.130-73.192	1.06-28.243	2.560–67.662
No. of measured, independent and observed [I> 2s(I)] reflections	4514, 777, 612	7182, 925, 865	67987, 6882, 6837
R_{int}	0.050	0.017	0.044
$R_1[I > 2\sigma(I)^{ab}$	0.0514	0.0264	0.0442
wR_2[I > 2$\sigma(I)$]^c	0.0906	0.0701	0.1258
GOF on$F^{2\ c}$	1.071	1.06	1.11

[a]This formula is the obtained by XR Single-crystal resolution, nevertheless, the formula of all the compounds was estimated through elemental analysis.

[b]$R_1 = [\sum(\|F_o| - |F_c\|)/\sum|F_o|]$.[c]w$R_2 = [\sum[w(F_o^2 - F_c^2)^2]/\sum[w(F_o^2)^2]]^{1/2}$Goodness-of-fit $S = [\Sigma\ [w(F_o^2–F_c^2)^2] / (n–p)]^{1/2}$.

Table S2: Distances between central mesoxalate carbon and its both coordinated central oxygens. X correspond to the label of the considered mesoxalate (L_A, L_B, L_C or L_D). The complete deprotonation of L_C results in a shorter C2C-O4C distance.

	Distance C2X-O3X(Å)	Distance C2X-O4X(Å)
Mesoxalate L_A	1.362 (5)	1.421 (5)
Mesoxalate L_B	1.381 (6)	1.416 (7)
Mesoxalate L_C	1.382 (6)	1.395 (6)
Mesoxalate L_D	1.387 (7)	1.419 (7)

Section 3. Magnetic Properties

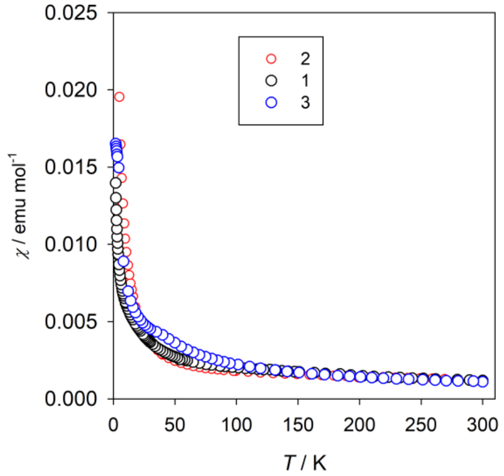

Figure S5. Magnetic susceptibility plot as a function of T for **1**-**3**. χ refers to the magnetic susceptibility per mole of Cu(II).

Section 4. DFT calculations

Compound 1

We have calculated the magnetic coupling constant between the Cu1 atoms across the alkoxido bridge, J_1, and the coupling between Cu1 and Cu2 across the *anti-anti* carboxylate bridge, J_2. For the calculation, we have taken six Cu atoms, six mesoxalate ligands, one chloride ion and six water molecules according to Figure S6. The Cu(II) ions are numbered according to the scheme in Figure S6b. The hydrogen atoms are not shown for clarity but were included in the calculation. The atomic positions are taken from the cif file, and the hydrogen atoms were added geometrically with the Gaussview 5.0, keeping the C_3 symmetry.

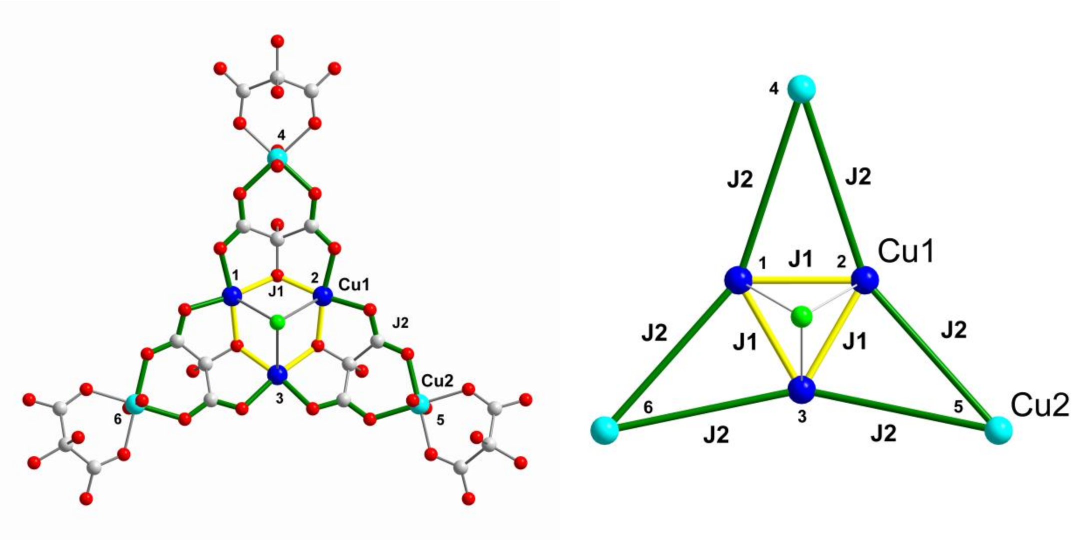

a) b)

Figure S6 a) Fragment of **1** used for the calculation of J_1 and J_2 (hydrogen atoms omitted for clarity). b) Schematic view of the magnetic exchange-pathways between Cu1 and Cu2 in **1**.

We calculated the energies of the E7, E3 and E1 broken-symmetry states HS |++++++>, MS |-++-++> and LS |+++---> with multiplicities 7, 3 and 1, respectively.

Table S3. Spin state of the Cu(II) ions, multiplicity and energy of the spin states for the calculus of J_1 and J_2 in **1**.

Spin State<br>|123456>	Spin Multiplicity<br>2S+1	Energy / H	Relative Energy/ cm^{-1}
HS |++++++>	7	E7=-14161.07505	460.02
MS |-++-++>	3	E3= -14161.07744	156.22
LS |+++--->	1	E1= -14161.07715	0

With those values we calculate the coupling constants J_1 and J_2 according to the following equations:

E1-E7 = $6J_2$ = -460.02 cm^{-1} J_2= -76.7 cm^{-1} (Equation S1)

E3-E7= $2J_1 + 4J_2$ =-523.93 cm^{-1} J_1 = -108.7 cm^{-1} (Equation S2)

The coupling through the alkoxido bridge, J_1, results in a value of -108.7 cm^{-1} and the coupling through the *anti-anti* carboxylate bridge J_2 = -76.7 cm^{-1}.

With the following fraction of the structure of **1**, which contains six Cu(II), six mesoxalate ligands and one Cl^-, we can calculate the magnetic coupling constants through the alkoxido bridge, J_1, and through the chloride bridge, J_3, Figure S7. The values are given below.

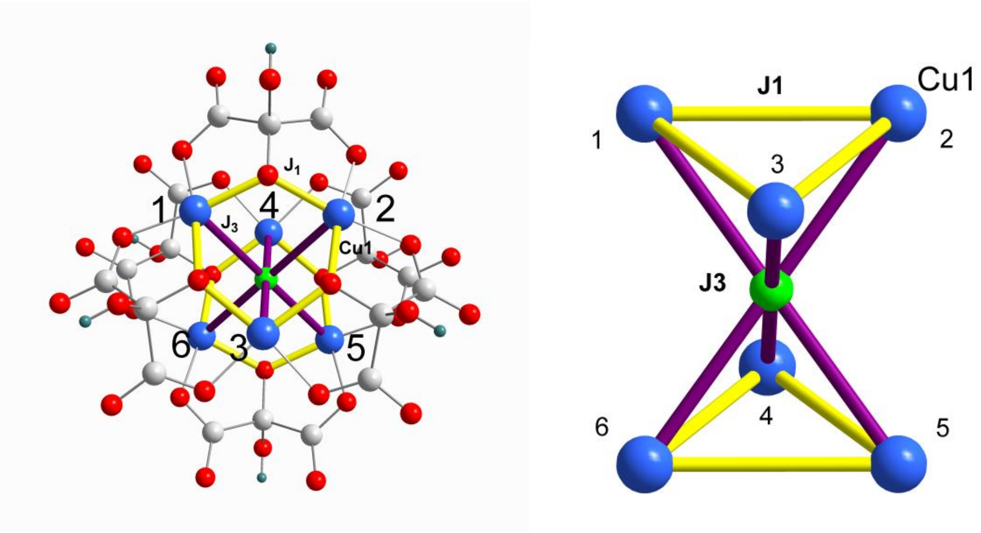

a) b)

Figure S7. a) Fragment of **1** with J_1 and J_3 magnetic exchange-pathways and Cu(II) ions' numbering scheme. b) Schematic view of a $Cu1_6$ cluster in **1** with J_1 and J_3 magnetic exchange-pathways.

Table S4. Spin state of the Cu(II) ions, multiplicity and energy of the spin states for the calculus of J_1 and J_3 in **1**.

Spin State<br><br>|123456>	Spin Multiplicity<br><br>2S+1	Energy / H	Relative Energy/ cm^{-1}
HS |++++++>	7	E7=-13701.93228	0.68
MS |-++-++>	3	E3= -13701.93406	-388.84
LS |+++--->	1	E1= -13701.93229	0

E1-E7= $3J_3$=-0.68 cm^{-1} J_3= -0.23 cm^{-1} (Equation S3)

E3-E7= $4J_1 + 2J_3$ = -389.52 cm^{-1} J_1= -97.3 cm^{-1} (Equation S4)

We find a slight difference in J_1 with respect to the previous calculation, but the difference is small (11cm^{-1}), which means that the method is correct and that the set of atoms is not critical for calculating the coupling constants.

Compound 2

We have calculated the J_1 and J_3 with the same set of atoms shown in Fig S6 with the atomic positions of compound **2**. Due to the almost identical structure of the Cu(II)/mesoxalate network, the values obtained are almost identical.

Table S5. Spin state of the Cu(II) ions, multiplicity and energy of the spin states for the calculus of J_1 and J_3 in **2**.

Spin State<br><br>|123456>	Spin Multiplicity<br><br>2S+1	Energy / H	Relative Energy/ cm^{-1}
HS |++++++>	7	E7=-13701.76707	0.64
MS |-++-++>	3	E3= -13701.76886	-392.71
LS |+++--->	1	E1= -13701.76707	0

E1-E7= $3J_3$= -0.64 cm^{-1} J_3= -0.21 cm^{-1} (Equation S5)

E3-E7= $4J_1 + 2J_3$ = -392.07 cm^{-1} J_1= -97.9 cm^{-1} (Equation S6)

Due to the almost identical structure, we assume that the value of J_2 is the same to that found for compound **1**.

Compound 3

The structure of **3** contains two different layers that we have labeled as *a* and *b*. *a* layers contain Cu1, Cu2 and Cu3 atoms (Figure 4) with a structure and connectivity similar to that of **1** and **2** with alkoxido-bridged trinuclear entities (J_1 and J_4) connected to copper(II) ions trough *anti-anti* carboxylate bridges (J_2 and J_3) with structural parameters and bridging modes that would lead to intralayer antiferromagnetic interactions, Figure. S8 [J_4 : Cu2OCu2 134.23(2)°; J_1 : Cu3OCu3 126.20(2)°, J_2 and J_3*anti-anti* carboxylate bridges connecting equatorial positions].

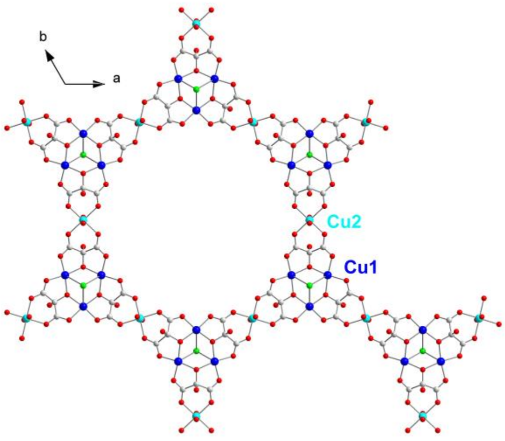

a)

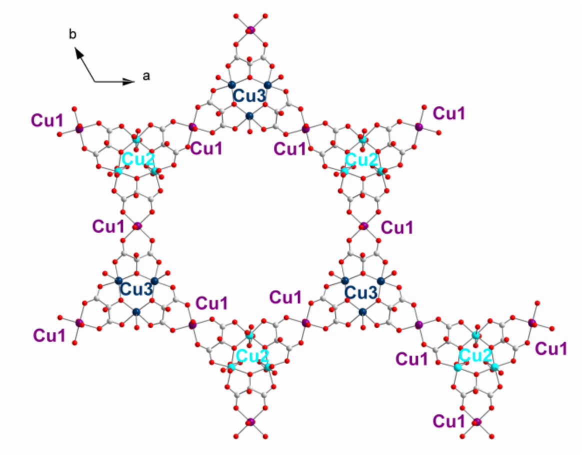

b)

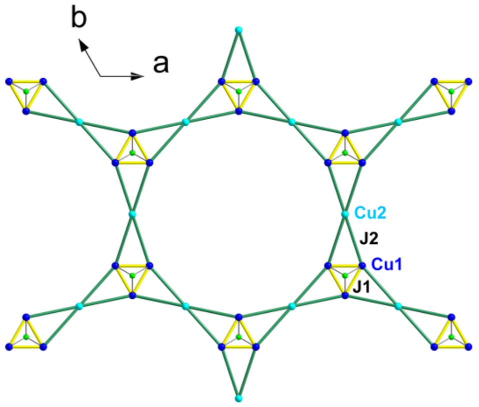

c)

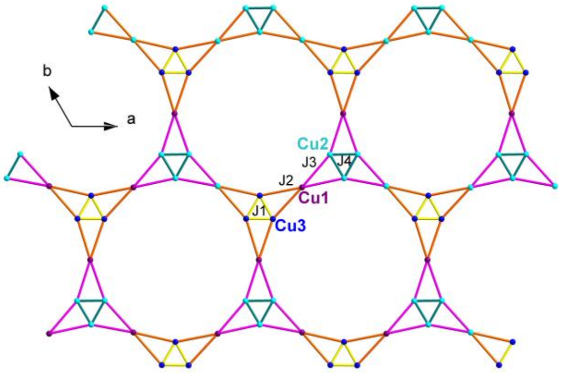

d)

Figure S8. a) view of the A layers in **1** b) view of the *a* layers in **3** c) magnetic coupling scheme in **1** with J_1 as the conection through the alkoxide bridge, J_2 as the conection through the *anti-anti*carboxilate bridge d) magnetic coupling scheme in the layers *a* in **3**. J_1 and J_4 coupling through alkoxido bridges, J_2 and J_3 coupling through the *anti-anti* carboxylate bridges. The layers a in **3** are less symmetric but with the same connectivity.

We have considered much more interesting the study of the *b* layers that have different connectivity in the carboxylate groups, Figure 7 and Figure S7. J_8 and J_5 refer to the couplings between Cu5 and Cu6 atoms through alkoxido bridges in the trinuclear entities; J_6 to the coupling between Cu6 and Cu4 through the *anti-syn* carboxylate bridge; and J_7 to the coupling between Cu4 and Cu5 through the *anti-anti* carboxylate bridge. J_6 is of special interest since it is the unique *anti-syn* bridge found in Cu(II) / mesoxalate complexes. Also, J_7 is singular since the mesoxalate adopts a μ_3-(κO:κO′,κO″:κO″,κO″:κO‴,κO⁗:κO″″) bridging mode with the two alcohol groups deprotonated.

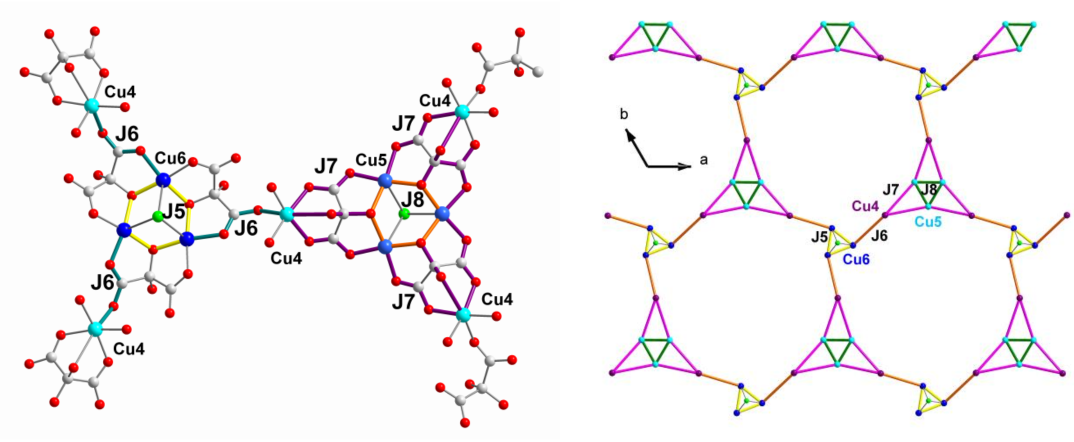

a) b)

Figure S9. a) A fragment of a *b* layer in **3** showing the connectivity. b) Schematic view of the *b* network with the magnetic coupling scheme.

Fort the calculus of J_7 and J_8 we have selected the following fragment that contains 6 Cu(II) ions, six mesoxalate ligands, one Cl^- and six coordination water molecules. The numbering of the Cu(II) ions is also shown.

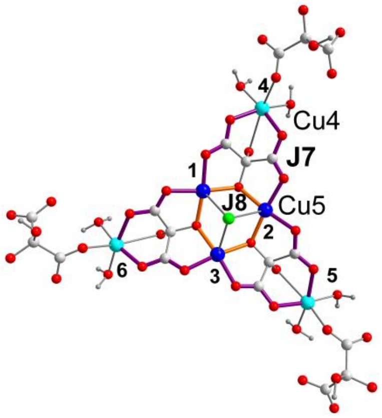

Figure S10. Fragment of **3** for the calculus of J_7 and J_8.

The carboxylate groups adopt an *anti-anti* connectivity and the magnetic coupling is reinforced by the participation of the two alkoxide groups that reduce the Cu5···Cu4 distance.

Table S6. Spin state of the Cu(II) ions, multiplicity and energy of the spin states for the calculus of J_7 and J_8 in **3**.

Spin State<br><br>|123456>	Spin Multiplicity<br><br>2S+1	Energy / H	Relative Energy/ cm^{-1}
HS |++++++>	7	E7=-14157.62869	852.83
MS |-+++-+>	3	E3= -14157.63134	271.07
LS |+++--->	1	E1= -14157.63258	0.00

E1-E7= $6J_7$ =-852 cm^{-1} J_7= -142 cm^{-1} (Equation S7)

E3-E7=$2J_8 + 4J_7$ = -581 cm^{-1} J_8= -6.9 cm^{-1} (Equation S8)

The coupling through the alkoxido bridge with a Cu5OCu5 angle of 118.69(2)º, J_8, gives a value of -6.9 cm^{-1}. And the coupling through the *anti-anti* carboxylate bridge results in a value of J_7= -142 cm^{-1}.

For the calculation of the magnetic coupling between Cu6 atoms through the alkoxido bridge, J_5, and the coupling between Cu6 and Cu4 atoms through the *anti-syn* carboxylate bridge, J_6, we have considered the following fragment. The values are given in Table S6.

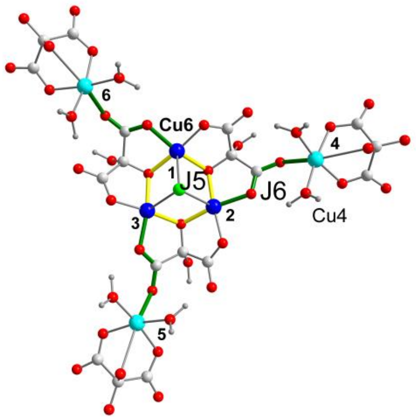

Figure S11. Fragment used for the calculation of the magnetic coupling constants J_5 and J_6.

Table S7. Spin state of the Cu(II) ions, multiplicity and energy of the spin states for the calculus of J_5 and J_6 in **3**.

Spin State<br>|123456>	Spin Multiplicity<br>2S+1	Energy / H	Relative Energy/ cm^{-1}
HS |++++++>	7	E7=-14157.71098	12.66
MS |-++-++>	3	E3= -14157.71104	0
LS |+++--->	1	E1= -14157.71047	125.32

E1-E7= $3J_6$ =112.66 cm^{-1} J_6= 37.6 cm^{-1} (Equation S9)

E3-E7=$2J_5$ = -12.66 cm^{-1} J_5= -6.3 cm^{-1} (Equation S10)

Finally we have calculated the magnetic coupling through the alkoxido, J_1, and alcohol-alkoxide groups, J_9, among Cu3 ions in compound **3**. The fragment used for this calculation is shown in Figure S10 and the values in the Table S7.

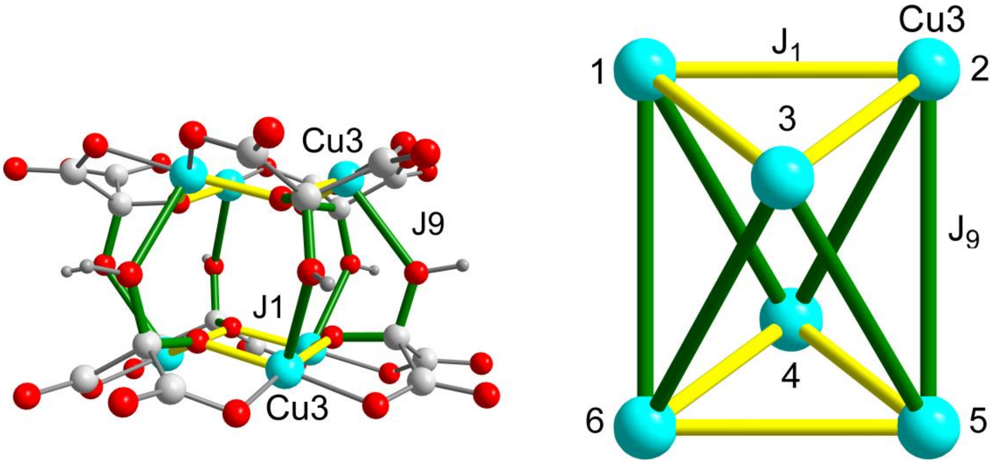

a) b)

Figure S12. a) fragment used for the calculation of J_1 and J_9. b) Magnetic coupling scheme between layers *a* in **3** among Cu3 ions through the alkoxide bridge, J_1 and through the alcohol-alkoxide bridge coupling, J_9. Note that the connectivity through the alcohol-alkoxide bridge is different from the Chloride bridge.

Table S8. Spin state of the Cu(II) ions, multiplicity and energy of the spin states for the calculus of J_1 and J_9 in **3**.

Spin State<br>|123456>	Spin Multiplicity<br>2S+1	Energy / H	Relative Energy/ cm^{-1}
HS |++++++>	7	E7= -13242.24222	250.82
MS |-+-+++>	3	E3= -13242.24337	0
LS |+++--->	1	E1= -13242.24223	249.92

$E1-E7= 6J_9 =-0.90\ cm^{-1}$ $J_9= -0.15\ cm^{-1}$ (Equation S11)

$E3-E7=2J_1 + 4J_9 = -250.82\ cm^{-1}$ $J_1= -125.1\ cm^{-1}$ (Equation S12)

Section 5: Proton conduction

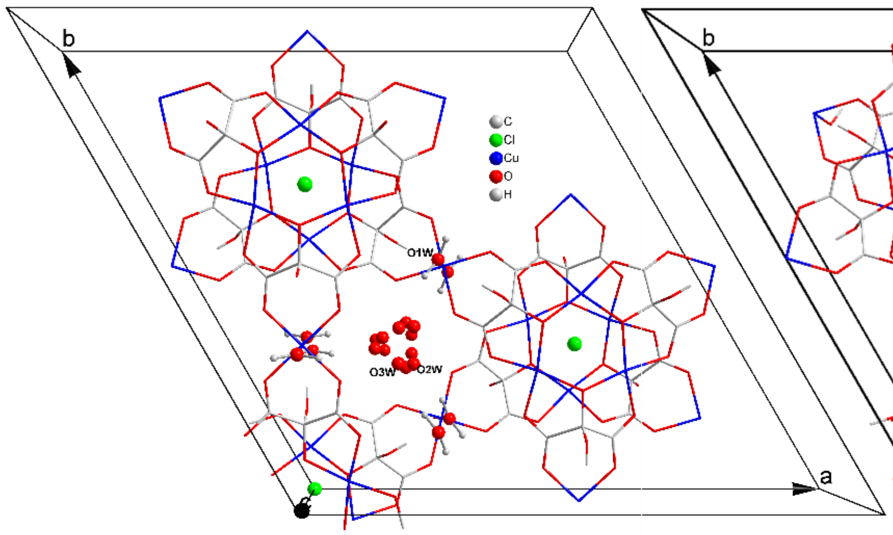

a)

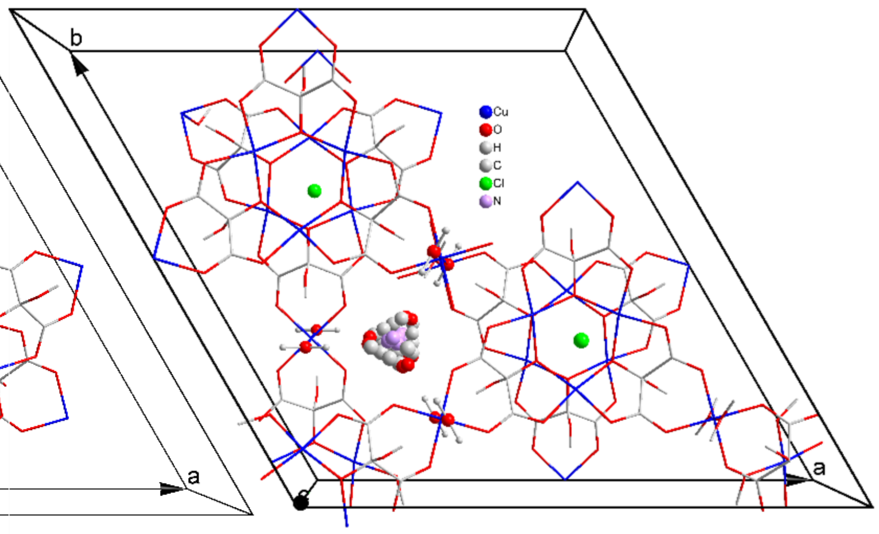

b)

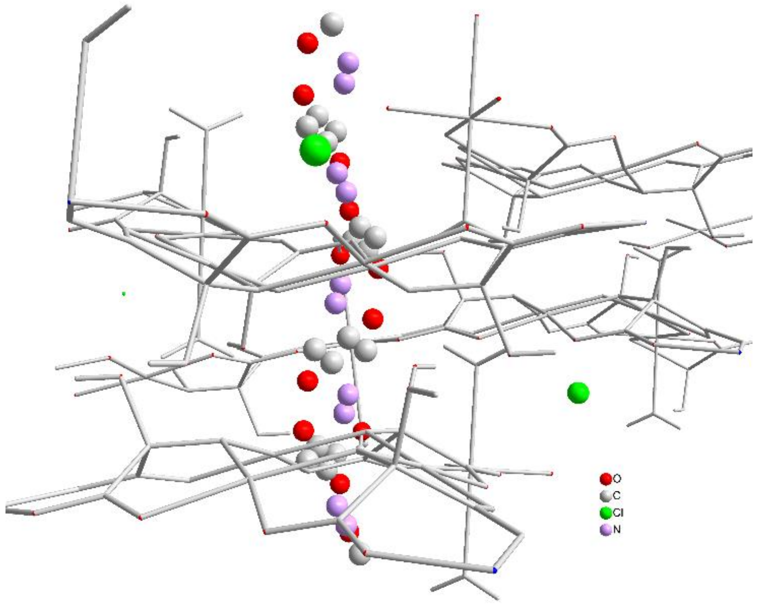

c)

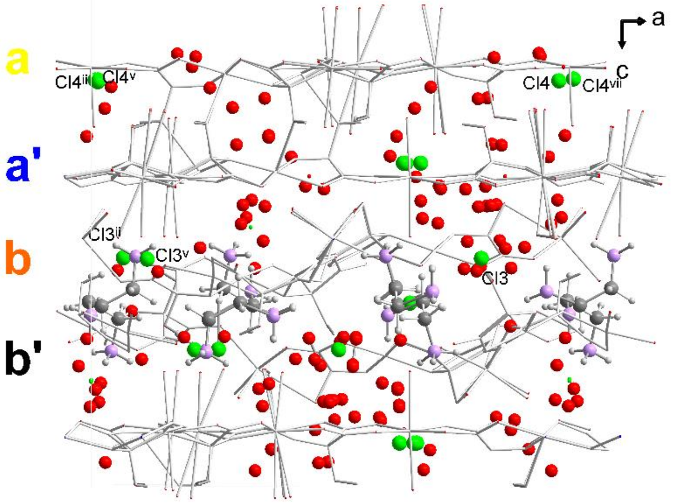

d)

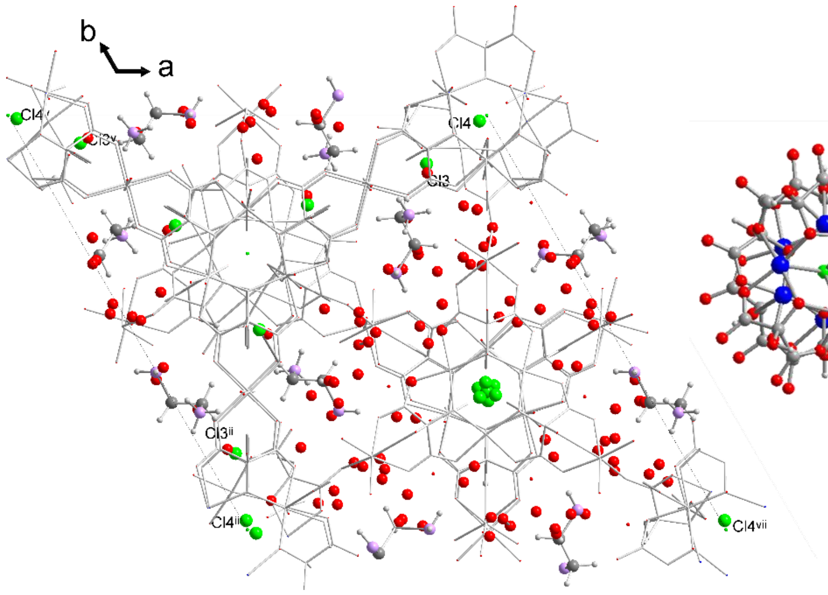

e)

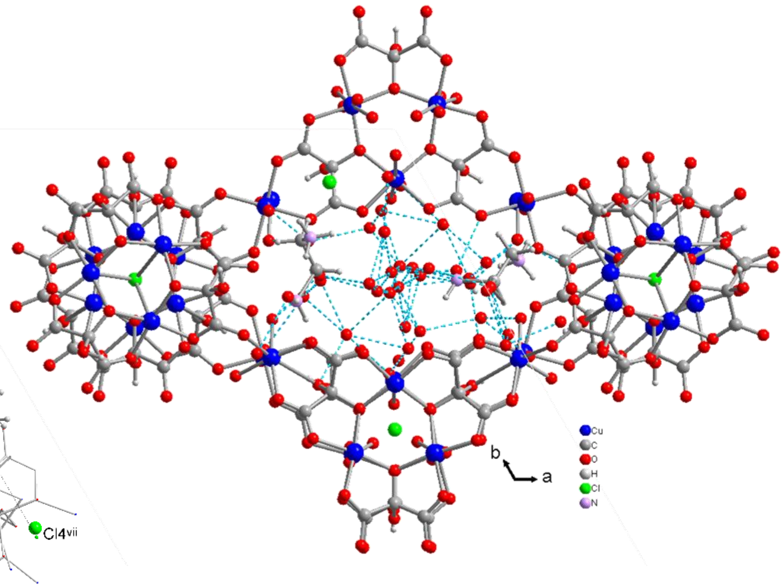

f)

Figure S13. Proton conduction medium for compounds **1**-**3**. a) View along *c* axis of compound **1**. Oxygen atoms of water molecules and/or hydronium cations are located disordered in the pores. For the seek of clarity, the anionic network has been represented as “wires or sticks”, except the coordination water molecules, O1W, and O2W and O3W located in the cavities along *c,* which are represented in “balls and sticks”; b) View along *c* axis for **2**. Crystallization water molecules are located in the cavities along *c* axis together with hydronium and dimethylammonium cations. Both can be found heavily disordered, and they have been refined with partial occupation. Hydrogen atoms could not be located. The bonds between N and C atoms of the dimethylammonium have been omitted for clarity; c) view along *b* axis of **2.** Disordered dimethylammonium cations and oxygen atoms of crystallization water molecules can be observed running across the cavity. Bonds between N and C atoms have been omitted for clarity; d) View along *b* axes for **3**. The anionic network has been displayed in grey for clarity. The oxygen atoms of crystallization water molecules and ethylenediammonium cations are located disordered in the pores, and they are represented as “ball and sticks”; e) Same as d, but view along *c* axis; f) View along *c* axis for the cavities of **3**, where water and ethylenediammonium cations are located. For the picture, only the stacking of layers *a*, *a’* and *b* has been used, for the seek of clarity. Although the water and cations are heavily disordered in the pores and any hydrogen-bonding pathway could be dilucidated, we have drawn the contacts between oxygen atoms of water molecules (depicted in dashed blue lines) with distances O-O between 2.7 and 3.3 Å, typical of donor-aceptor distances in hydrogen bonding, indicating that there is a path for proton conduction.

Section 6. Main Distances and Angles

Table S9. Selected bond lengths (Å) and angles (°) for **1**

Distances (Å)			
Cu1—O1	1.940 (3)	Cu2—O2^{v}	1.945 (3)
Cu1—O1^{ii}	1.941 (3)	Cu2—O2	1.945 (3)
Cu1—O3^{iii}	1.9434 (18)	Cu2—O2^{vi}	1.945 (3)
Cu1—O3	1.9434 (18)	Cu2—O1W	2.383 (6)
Cu2—O2^{iv}	1.945 (3)	Cu2—O1W^{iv}	2.384 (6)
Angles (°)			
C1^{vii}—C2—C1^{viii}	110.8 (5)	O2—Cu2—O2^{vi}	92.85 (17)
O1—Cu1—O1^{ii}	86.97 (18)	O2^{iv}—Cu2—O1W	90.08 (15)
O1—Cu1—O3^{iii}	84.71 (14)	O2^{v}—Cu2—O1W	90.08 (15)
O1^{ii}—Cu1—O3^{iii}	171.66 (14)	O2—Cu2—O1W	89.92 (15)
O1—Cu1—O3	171.65 (14)	O2^{vi}—Cu2—O1W	89.92 (15)
O1^{ii}—Cu1—O3	84.70 (14)	O2^{iv}—Cu2—O1W^{iv}	89.91 (15)
O3^{iii}—Cu1—O3	103.6 (2)	O2^{v}—Cu2—O1W^{iv}	89.91 (15)
O2^{iv}—Cu2—O2^{v}	92.85 (17)	O2—Cu2—O1W^{iv}	90.08 (15)
O2^{iv}—Cu2—O2	180.0	O2^{vi}—Cu2—O1W^{iv}	90.09 (15)
O2^{v}—Cu2—O2	87.15 (17)	O3^{i}—C2—C1^{vii}	110.5 (3)
O2^{iv}—Cu2—O2^{vi}	87.15 (17)	O1W—Cu2—O1W^{iv}	180.0
O2^{v}—Cu2—O2^{vi}	180.0	Cu1—O3—Cu1^{ix}	124.1 (2)

Symmetry codes: (i) *x-y*, *x*, *-z*+1; (ii) *-x*+*y*, *y*, *z*; (iii) *-y*, *x-y*, *z*; (iv) *-x*-1/3, *-y*+1/3, *-z*+1/3; (v) *y*-1/3, *x*+1/3, *-z*+1/3; (vi) *-y*, *-x*, *z*; (vii) *y*, *-x*+*y*, *-z*+1; (viii) *-x*, *-x*+*y*, *-z*+1; (ix) *-x*+*y*, *-x*, *z*.

Table S10. Selected bond lengths (Å) and angles (°) for **2**

Distances (Å)			
Cu1—O3	1.9412 (16)	Cu2—O1^{iii}	1.9462 (18)
Cu1—O3^{i}	1.9413 (16)	Cu2—O1^{iv}	1.9462 (18)
Cu1—O2	1.9415 (10)	Cu2—O1^{v}	1.9462 (18)
Cu1—O2^{ii}	1.9415 (10)	Cu2—O1W	2.414 (4)
Cu2—O1	1.9462 (18)	Cu2—O1W^{v}	2.414 (4)
Angles (°)			
O3—Cu1—O3^{i}	86.96 (10)	O1—Cu2—O1W	90.25 (9)
O3—Cu1—O2	171.69 (8)	O1^{iii}—Cu2—O1W	90.26 (9)
O3^{i}—Cu1—O2	84.75 (8)	O1^{iv}—Cu2—O1W	89.74 (9)
O3—Cu1—O2^{ii}	84.75 (8)	O1^{v}—Cu2—O1W	89.74 (9)
O3^{i}—Cu1—O2^{ii}	171.69 (8)	O1—Cu2—O1W^{v}	89.75 (9)
O2—Cu1—O2^{ii}	103.53 (12)	O1^{iii}—Cu2—O1W^{v}	89.74 (9)
O1—Cu2—O1^{iii}	92.28 (10)	O1^{iv}—Cu2—O1W^{v}	90.26 (9)
O1—Cu2—O1^{iv}	87.72 (10)	O1^{v}—Cu2—O1W^{v}	90.25 (9)

O1^{iii}—Cu2—O1^{iv}	180.0	O1W—Cu2—O1W^{v}	180.0 (2)
O1—Cu2—O1^{v}	180.0	Cu1^{vi}—O2—Cu1	124.18 (12)
O1^{iii}—Cu2—O1^{v}	87.72 (10)	Cu2—O1W—H1W	115.5 (18)
O1^{iv}—Cu2—O1^{v}	92.28 (10)		

Symmetry codes: (i) -*x*+*y*, *y*, *z*; (ii) -*x*+*y*, -*x*+1, *z*; (iii) *x*, *x*-*y*+1, *z*; (iv) -*x*, -*x*+*y*, -*z*+2; (v) -*x*, -*y*+1, -*z*+2; (vi) -*y*+1, *x*-*y*+1, *z*.

Table S11. Selected bond lengths (Å) and angles (°) for **3**

Distances (Å)			
Cu1—O2B	1.956 (4)	Cu3—O1A	1.960 (4)
Cu1—O6B	1.964 (4)	Cu3—O4A^{iii}	2.483 (3)
Cu1—O6A	1.983 (4)	Cu4—O3W	1.952 (5)
Cu1—O2A	1.992 (4)	Cu4—O4W	1.961 (5)
Cu1—O1W	2.328 (5)	Cu4—O6C	1.971 (5)
Cu1—O2W	2.328 (5)	Cu4—O2C	1.977 (5)
Cu2—O3B	1.923 (4)	Cu4—O2D	2.262 (4)
Cu2—O5B^{i}	1.927 (5)	Cu4—O4C	2.620 (4)
Cu2—O3B^{i}	1.935 (5)	Cu5—O5C^{iv}	1.929 (4)
Cu2—O1B	1.959 (5)	Cu5—O1C	1.935 (5)
Cu2—O5W'	2.321 (11)	Cu5—O3C^{iv}	1.937 (4)
Cu2—O5W	2.610 (13)	Cu5—O3C	1.958 (5)
Cu2—O6W'	2.64 (2)	Cu6—O5D^{ii}	1.924 (4)
Cu2—O6W	2.70 (3)	Cu6—O3D	1.943 (4)
Cu3—O3A^{ii}	1.930 (4)	Cu6—O1D	1.950 (4)
Cu3—O3A	1.935 (4)	Cu6—O3D^{ii}	1.970 (4)
Cu3—O5A^{ii}	1.940 (4)	Cu6—Cl1	2.6919 (16)
Angles (°)			
O2B—Cu1—O6B	92.31 (15)	O3A—Cu3—O1A	84.35 (14)
O2B—Cu1—O6A	175.50 (19)	O5A^{ii}—Cu3—O1A	90.61 (13)
O6B—Cu1—O6A	87.60 (16)	O3A^{ii}—Cu3—O4A^{iii}	99.77 (13)
O2B—Cu1—O2A	88.56 (16)	O3A—Cu3—O4A^{iii}	97.76 (13)
O6B—Cu1—O2A	173.87 (19)	O5A^{ii}—Cu3—O4A^{iii}	82.59 (14)
O6A—Cu1—O2A	91.05 (14)	O1A—Cu3—O4A^{iii}	80.08 (14)
O2B—Cu1—O1W	95.43 (19)	O3W—Cu4—O4W	92.47 (17)
O6B—Cu1—O1W	94.76 (19)	O3W—Cu4—O6C	88.4 (2)
O6A—Cu1—O1W	89.05 (17)	O4W—Cu4—O6C	172.1 (2)
O2A—Cu1—O1W	91.20 (18)	O3W—Cu4—O2C	166.9 (2)
O2B—Cu1—O2W	87.18 (19)	O4W—Cu4—O2C	88.13 (19)
O6B—Cu1—O2W	86.17 (19)	O6C—Cu4—O2C	89.23 (15)
O6A—Cu1—O2W	88.33 (16)	O3W—Cu4—O2D	95.8 (2)
O2A—Cu1—O2W	87.81 (17)	O4W—Cu4—O2D	98.34 (18)

O1W—Cu1—O2W	177.18 (15)	O6C—Cu4—O2D	89.36 (19)
O3B—Cu2—O5B^{i}	176.09 (19)	O2C—Cu4—O2D	97.05 (18)
O3B—Cu2—O3B^{i}	100.05 (19)	O3W—Cu4—O4C	92.98 (19)
O5B^{i}—Cu2—O3B^{i}	83.85 (19)	O4W—Cu4—O4C	98.47 (17)
O3B—Cu2—O1B	83.83 (18)	O6C—Cu4—O4C	73.66 (18)
O5B^{i}—Cu2—O1B	92.34 (18)	O2C—Cu4—O4C	74.00 (17)
O3B^{i}—Cu2—O1B	171.7 (3)	O2D—Cu4—O4C	160.65 (14)
O3B—Cu2—O5W'	92.0 (3)	O5C^{iv}—Cu5—O1C	89.32 (15)
O5B^{i}—Cu2—O5W'	86.7 (4)	O5C^{iv}—Cu5—O3C^{iv}	85.13 (16)
O3B^{i}—Cu2—O5W'	104.5 (3)	O1C—Cu5—O3C^{iv}	173.53 (18)
O1B—Cu2—O5W'	82.6 (4)	O5C^{iv}—Cu5—O3C	173.35 (17)
O3B—Cu2—O5W	85.7 (4)	O1C—Cu5—O3C	85.74 (17)
O5B^{i}—Cu2—O5W	94.5 (4)	O3C^{iv}—Cu5—O3C	99.5 (2)
O3B^{i}—Cu2—O5W	84.9 (4)	O5D^{ii}—Cu6—O3D	174.89 (17)
O1B—Cu2—O5W	102.8 (4)	O5D^{ii}—Cu6—O1D	90.68 (17)
O3B—Cu2—O6W'	88.1 (5)	O3D—Cu6—O1D	84.74 (16)
O5B^{i}—Cu2—O6W'	92.8 (5)	O5D^{ii}—Cu6—O3D^{ii}	85.48 (17)
O3B^{i}—Cu2—O6W'	81.2 (6)	O3D—Cu6—O3D^{ii}	98.5 (2)
O1B—Cu2—O6W'	91.6 (6)	O1D—Cu6—O3D^{ii}	166.20 (18)
O5W'—Cu2—O6W'	174.2 (6)	O5D^{ii}—Cu6—Cl1	97.77 (13)
O3B—Cu2—O6W	88.9 (8)	O3D—Cu6—Cl1	85.84 (12)
O5B^{i}—Cu2—O6W	91.0 (8)	O1D—Cu6—Cl1	108.34 (13)
O3B^{i}—Cu2—O6W	95.3 (9)	O3D^{ii}—Cu6—Cl1	85.33 (12)
O1B—Cu2—O6W	77.3 (9)	Cu3^{v}—O3A—Cu3	126.23 (16)
O5W—Cu2—O6W	174.5 (9)	Cu2—O3B—Cu2^{vi}	134.23 (19)
O3A^{ii}—Cu3—O3A	101.24 (16)	Cu5^{vii}—O3C—Cu5	118.68 (17)
O3A^{ii}—Cu3—O5A^{ii}	83.78 (14)	Cu6—Cl1—Cu6^{ii}	74.06 (5)
O3A—Cu3—O5A^{ii}	174.80 (14)	Cu6—Cl1—Cu6^{v}	74.06 (5)
O3A^{ii}—Cu3—O1A	174.35 (14)	Cu6^{ii}—Cl1—Cu6^{v}	74.06 (5)

Symmetry codes: (i) -*y*+2, *x*-*y*+1, *z*; (ii) -*x*+*y*, -*x*+1, *z*; (iii) *y*-1/3, -*x*+*y*+1/3, -*z*+4/3; (iv) -*y*+1, *x*-*y*, *z*; (v) -*y*+1, *x*-*y*+1, *z*; (vi) -*x*+*y*+1, -*x*+2, *z*; (vii) -*x*+*y*+1, -*x*+1, *z*;
